# Fine‐scale oceanographic drivers of reef manta ray (*Mobula alfredi*) visitation patterns at a feeding aggregation site

**DOI:** 10.1002/ece3.7357

**Published:** 2021-03-24

**Authors:** Joanna L. Harris, Phil Hosegood, Edward Robinson, Clare B. Embling, Simon Hilbourne, Guy M. W. Stevens

**Affiliations:** ^1^ The Manta Trust Dorset UK; ^2^ School of Biological and Marine Sciences University of Plymouth Plymouth UK

**Keywords:** acoustic telemetry, boosted regression trees, cold‐water bores, foraging ecology, internal waves, Langmuir Circulation

## Abstract

Globally, reef manta rays (*Mobula alfredi*) are in decline and are particularly vulnerable to exploitation and disturbance at aggregation sites. Here, passive acoustic telemetry and a suite of advanced oceanographic technologies were used for the first time to investigate the fine‐scale (5‐min) influence of oceanographic drivers on the visitation patterns of 19 tagged *M. alfredi* to a feeding aggregation site at Egmont Atoll in the Chagos Archipelago. Boosted regression trees indicate that tag detection probability increased with the intrusion of cold‐water bores propagating up the atoll slope through the narrow lagoon inlet during flood tide, potentially transporting zooplankton from the thermocline. Tag detection probability also increased with warmer near‐surface temperature close to low tide, with near‐surface currents flowing offshore, and with high levels of backscatter (a proxy of zooplankton biomass). These combinations of processes support the proposition that zooplankton carried from the thermocline into the lagoon during the flood may be pumped back out through the narrow inlet during an ebb tide. These conditions provide temporally limited feeding opportunities for *M. alfredi*, which are tied on the tides. Results also provide some evidence of the presence of Langmuir Circulation, which transports and concentrates zooplankton, and may partly explain why *M. alfredi* occasionally remained at the feeding location for longer than that two hours. Identification of these correlations provides unique insight into the dynamic synthesis of fine‐scale oceanographic processes which are likely to influence the foraging ecology of *M. alfredi* at Egmont Atoll, and elsewhere throughout their range.

## Graphical abstract

A dynamic synthesis of fine‐scale oceanographic processes influences the foraging ecology of zooplanktivorous *Mobula alfredi* at Egmont Atoll in the Chagos Archipelago; British Indian Ocean Territory. These processes indicate that internal waves and cold‐water bores potentially transport zooplankton from the thermocline providing temporally limited feeding opportunities which are enhanced by the tide. Furthermore, processes such as Langmuir Circulation may provide extended foraging opportunities by transporting and concentrating zooplankton in the region.

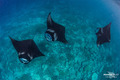

## 1 INTRODUCTION

Reef manta rays (*Mobula alfredi*) are large zooplanktivorous elasmobranchs of the family Mobulidae (Hosegood et al., 2020; Marshall et al., 2009; White et al., 2017). The global population is widely distributed in highly fragmented subpopulations throughout tropical and sub‐tropical waters of the Indo‐West Pacific Oceans (Couturier et al., 2012; Marshall et al., 2019). Subpopulations appear to have limited home ranges, typically centered around coral reef ecosystems (Couturier et al., 2018; Kessel et al., 2017; McCauley et al., 2014; Setyawan et al., 2018). Aggregation behavior is characteristic of the species, whereby subpopulations will concentrate the majority of their activities at certain “hotspot” locations (Couturier et al., 2018; Harris et al., 2020; Setyawan et al., 2018). These aggregations typically occur within particular discrete habitats (Harris et al., 2020; Stevens, 2016) such as cleaning stations (O’Shea et al., 2010), and locations where they engaged in social (Perryman et al., 2019; Stevens, 2016) or reproductive activities (Stevens et al., 2018). Large feeding aggregations also occur and are often associated with the species’ reliance on dense assemblages of prey (Armstrong et al., 2016) in a largely oligotrophic environment (Morel et al., 2010).

Extensive targeted and bycatch fisheries of *M. alfredi*, driven in part for their gill plates [prebranchial appendages, used to filter their zooplankton prey from the water (Paig‐Tran et al., 2013), which are utilized in the Asian medicinal trade (O’Malley et al., 2017)], have led to dramatic subpopulation declines in recent decades (Couturier et al., 2013; Lawson et al., 2017; Marshall et al., 2019; Rohner et al., 2017). Population recovery from such exploitation is hindered by their conservative life‐history traits; the species are slow‐growing, late to mature, and only have a few offspring in their lifetime (Dulvy et al., 2014; Stevens, 2016).

*Mobula alfredi* are particularly vulnerable to exploitation and changes in climate at feeding aggregation sites. For example, anthropogenic disturbance may reduce individual *M. alfredi* fitness by driving them away from productive feeding areas (Murray et al., 2019; Venables et al., 2016), which has been highlighted as a major conservation concern for the species (Harris et al., 2020; Murray et al., 2019). Feeding behavior may also be disrupted by enhanced stratification driven by rising sea surface temperatures, which can decrease marine phytoplankton (Roxy et al., 2016), and with it zooplankton biomass (Richardson, 2008).

Studies which investigate *M. alfredi* aggregation behavior have associated their occurrence with various broadscale physical factors, such as wind speed, moon phase, sea surface temperature, and tidal phase (Couturier et al., 2018; Dewar et al., 2008; Jaine et al., 2012; Peel, Stevens, et al., 2019). However, the fine‐scale changes in the oceanographic environment that potentially drive feeding aggregations have yet to be investigated.

**FIGURE 1:**
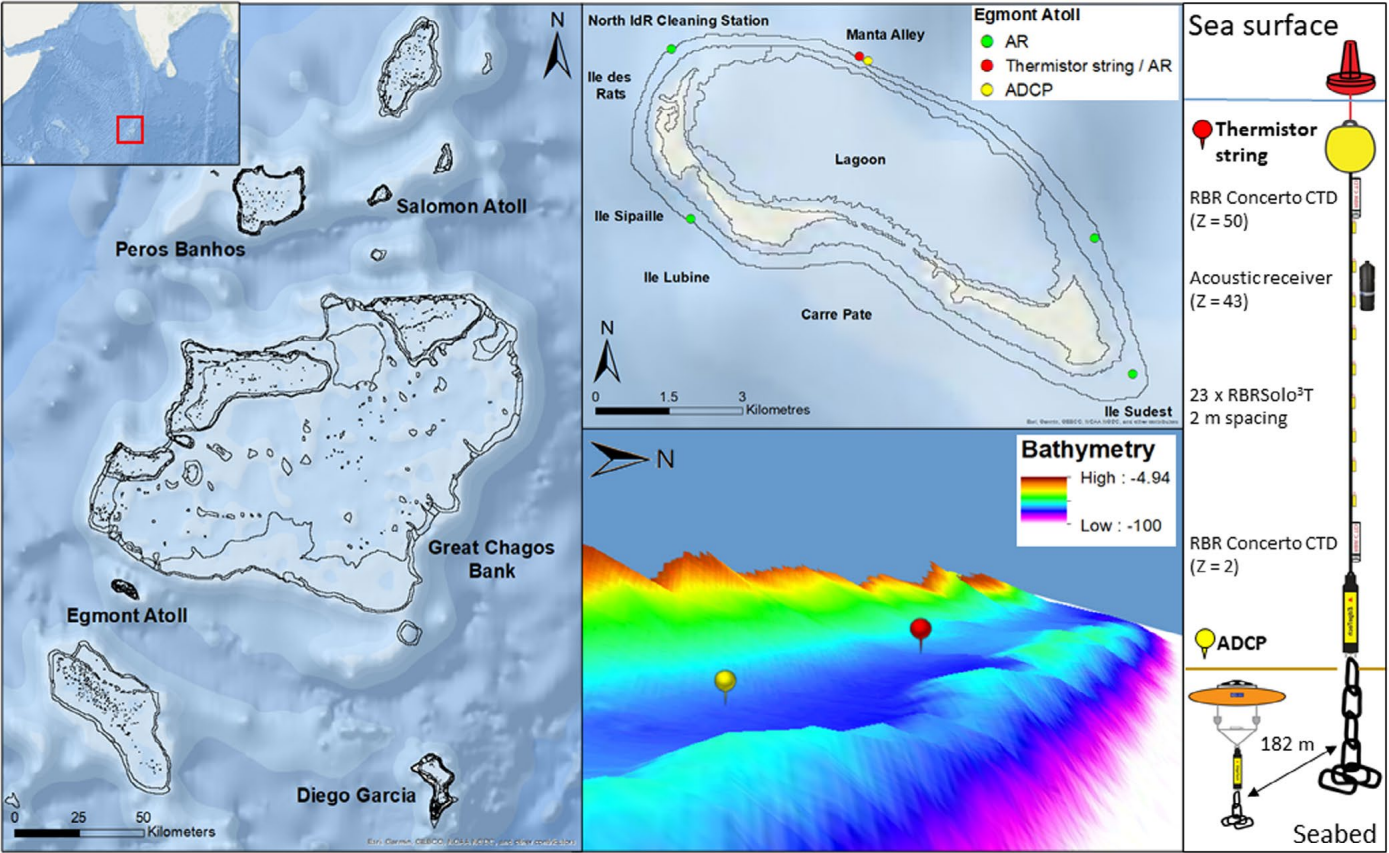
The Central Indian Ocean with Chagos Archipelago; British Indian Ocean Territory indicated within the red box (left inset). The Chagos Archipelago with Egmont Atoll indicated within the red box (left). Egmont Atoll and the location of the oceanographic and acoustic receiver mooring in Manta Alley (red and yellow dots) and four acoustic receivers (green dots) (top right). Bathymetric view of Manta Alley obtained via multibeam survey (E. Robinson, P. Hosegood, A. Bolton, unpublished data) showing the location of the moorings (bottom right). Bottom right legend showing instrument configurations of the long thermistor string (red dot/pin) and subsurface ADCP moorings (yellow dot/pin) deployed 182 m apart anchored at a depth of 66 m. Z is the height above the seabed

Situated in the central Indian Ocean, the Chagos Archipelago (Figure 1) has been uninhabited for many decades (excluding Diego Garcia Atoll; Sheppard et al., 2012). Due to the lack of human influence, such as coastal development and anthropogenic pollution, the region is considered virtually pristine (Readman et al., 2013). Owing to the region's unique marine environment, a no‐take marine protected area (MPA), which encompasses the entire exclusive economic zone (EEZ; 640,000 km^2^) except for a 3 nm exclusion around the boundary of Diego Garcia Atoll, was established in 2010 (Sheppard et al., 2012). The archipelago supports a subpopulation of *M*. *alfredi* which is largely undocumented due to the remoteness of the location and strict protective measures; as is the region's physical oceanographic environment (Hosegood et al., 2019). Broadscale studies conducted in the region indicated that Egmont Atoll, situated in the southwest of the archipelago, provides key habitats for this *M. alfredi* subpopulation (Andrzejaczek et al., 2020; Harris, 2019). Feeding *M. alfredi* are regularly observed around the atoll (Harris, 2019), behavior which is thought to be associated with shallow bathymetry, low current speeds, and cooler sea surface temperatures (Armstrong et al., 2016; Couturier et al., 2018; Harris, 2019; Jaine et al., 2012; Peel, Stevens, et al., 2019). Together, these factors may act to induce upwelling of nutrients, increasing primary and secondary production (McManus et al., 2005). Furthermore, currents interacting with topography may aggregate zooplankton (Genin et al., 2005), resulting in highly productive feeding grounds for a range of species, including *M. alfredi* (Hosegood et al., 2019).

Field observations have identified an *M. alfredi* feeding aggregation “hotspot” at the north of Egmont Atoll (J. Harris and G. Stevens, unpublished data). However, *M. alfredi* feeding activity can be dramatically different from one day to another, with little apparent change in broadscale oceanographic conditions (Harris, 2019). Therefore, a greater understanding of how *M. alfredi* respond to fine‐scale environmental drivers is needed. Here, passive acoustic telemetry and in situ oceanographic monitoring are used to investigate *M. alfredi* activity at Egmont Atoll, and assess what physical factors drive fine‐scale (5‐min) visitation patterns at the observed feeding aggregation hotspot. This study aims to enhance the current understanding of *M. alfredi* foraging ecology by providing detailed insight into their fine‐scale movement patterns in response to natural changes in their oceanographic environment.

## 2 METHODS

### 2.1 Study site

**FIGURE 2:**
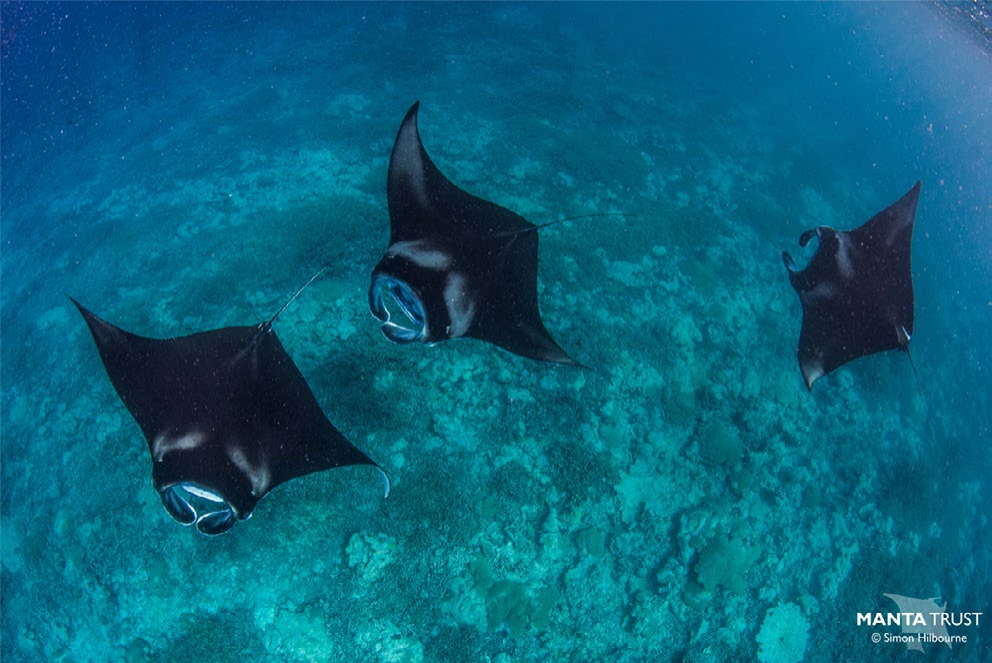
Reef manta rays (*Mobula alfredi*) engaged in feeding activities at the Manta Alley feeding aggregation site in north Egmont Atoll. Photo by Simon Hilbourne, Manta Trust

The Chagos Archipelago is comprised of seven atolls, several large submerged banks, and more than 60 low lying islands, located at the southernmost end of the Lakshadweep–Maldives–Chagos ridge; 450 km south of the Maldives (Sheppard et al., 2012; Figure 1). Egmont Atoll's geomorphology is typical of an atoll with an interior lagoon system which is separated from the open ocean by reef crests and flats, with narrow connecting channel systems (Woodroffe & Biribo, 2011). During six expeditions, between January 2015 and December 2019 (J. Harris and G. Stevens, unpublished data), the authors repeatedly observed aggregations of *M. alfredi* engaged in feeding activity at a site called Manta Alley (Figure 1). At this foraging hotspot, using in‐water observations (Figure 2) and ROVs, *M. alfredi* were recorded feeding at the surface and down to a depth of 120 m (J. Harris and G. Stevens, unpublished data; C. Diaz and N. Foster, unpublished data). Manta Alley is located 100 m north of Egmont Atoll's northeast rim, where two narrow (<350 m) passages are situated. From the shallow lagoon passages (<5 m), the topography slopes steeply down (up to 47°) before reaching a 50 m wide plateau 80 m from the lagoon, with a depth of 65–71 m. On the seaward side, there is a narrow ridge which inclines steeply (up to 39°) to a height of approximately 10 m, followed by another sharp slope down to >100 m (E. Robinson, P. Hosegood, A. Bolton, unpublished data; Figure 1).

### 2.2 Oceanographic moorings

Two instrumented oceanographic moorings were deployed in Manta Alley (Figure 1) from a research ship on 30th December 2019. Both moorings were positioned within Manta Alley. The first was a subsurface taut‐line mooring deployed in 66 m, with the uppermost buoyancy element at a depth of 20 m. Temperature was measured by RBRSolo^3^T temperature sensors positioned at 2 m intervals from 4 to 48 m above the seabed. In addition to the temperature sensors, RBR Concerto conductivity–temperature–depth (CTD) sensors with a sampling interval of 5 s were positioned at 2 and 50 m above the bed. An acoustic receiver (see *acoustic receiver array* section below) was positioned approximately 7 m below the near‐surface CTD, at 43 m above the bed. The second mooring, deployed 182 m southeast of the first, comprised an upward‐facing Nortek Signature 500 kHz acoustic Doppler current profiler (ADCP), mounted on a subsurface buoy 3 m above the seabed. Both moorings were recovered on 17th March 2020; however, the Nortek Signature 500 kHz ADCP had ceased sampling on 10th March 2020.

### 2.3 Acoustic tag deployment

Tagging activities were carried out at Egmont Atoll between November 19, 2019 and December 3, 2019 while freediving. Twenty VEMCO V16‐4x acoustic transmitter tags (Vemco Inc.), each tethered to a titanium anchor (Wildlife Computers) with a small diameter cable, were deployed on the right dorsal musculature using a modified Hawaiian hand sling while swimming behind the *M. alfredi*. Each tag was set to operate at 69 kHz and transmit a unique acoustic signal at random intervals between 30 and 90 s. Before being tagged, the ventral side of each *M. alfredi* was photographed to capture their unique spot pattern for identification purposes (Marshall & Pierce, 2012), and their sex and size class (a proxy of maturity status) were recorded (Stevens, 2016). Five of the twenty *M. alfredi* that were tagged were re‐sighted at their tagging locations between three and 12 days after deployment. All five were observed to be engaged in normal feeding activities (Stevens, 2016). All activities were approved by the University of Plymouth Animals in Science Ethics Committee under permit ETHICS‐24‐2019.

### 2.4 Acoustic receiver array

An acoustic array of five VR2W‐69 kHz omnidirectional acoustic receivers (Vemco Inc.) was deployed at depths ranging from 12 to 22 m below the sea surface on the reef flat close to the reef slope at sites corresponding to known *M. alfredi* aggregation areas around the outer rim of Egmont Atoll (Figure 1). Four of the receivers were suspended approximately 2 m above the seabed, while the fifth was attached to an oceanographic mooring 43 m above the seabed at the Manta Alley aggregation site. Acoustic tags were detected within approximately 160 m of the receivers: mean = 162 ± 31 m (*SD*) as determined by range testing conducted following the method described by Lea (2017).

### 2.5 Acoustic tag analysis

All tag detection data were imported into VUE software (version 2.6.2) and filtered for active tags. The False Detection Analyser (VUE version 2.6.2) was then used to identify false detections, whereby the ratio of short and long periods between detections is calculated from the time between detections on each receiver (Simpfendorfer et al., 2015). Here, the default short to long periods of <30 min and >12 hr, respectively, were used (Simpfendorfer et al., 2015) and all detections suspected to be false were removed from analysis. The percentage of sightings at each location was then projected in ArcGIS 10.7.

To assess whether Egmont Atoll can be considered a key habitat, residency indices (RI) were calculated using the following form (Peel, Stevens, et al., 2019), allowing comparison of residency patterns at Egmont Atoll between *M. alfredi* regardless of differences in tracking periods (Daly et al., 2014).

$$\text{RI}\;\text{(\% )}=\frac{\text{Number}\;\text{of}\;\text{days}\;\text{detected}}{\text{Number}\;\text{of}\;\text{days}\;\text{between}\;\text{first}\;\text{and}\;\text{last}\;\text{detection}}\times 100.$$

To assess the intensity at which locations were utilized, the amount of time each tagged *M. alfredi* spent within the detection range of each acoustic receivers was calculated using the VTrack R package (Campbell et al., 2012) in R 3.5.2 (R Core Team, 2018). Briefly, each tag detection was classed as a resident or nonresident events. A resident event began when there were two or more successive detections (Nalesso et al., 2019) at the same receiver within 60 min. Termination of the resident event occurred at the time of the last detection when there were no further detections within 60 min, or when the tag was detected at least twice at another receiver (Campbell et al., 2012; Nalesso et al., 2019).

### 2.6 Environmental influences: boosted regression trees

Boosted regression trees (BRT) were used to investigate the relationship between environmental variables and the visitation patterns of tagged *M. alfredi* to the feeding aggregation site at Manta Alley. The modeling technique is based on two algorithms: regression trees models and boosting, which build and combine large numbers of relatively small trees by fitting each new tree to the residuals of the last (Elith et al., 2008). Each tree is constructed through a series of binary splits of predictor variables (Hastie et al., 2009), which occur based on the homogeneity of their relationship to the response variable (Colin et al., 2017). Multiple splits are tested, and partitioning occurs when the greatest improvement of homogeneity is found (Colin et al., 2017). Advantages of this modeling technique include its ability to fit complex, nonlinear relationships, model interactions between response variables (Elith et al., 2008), and the appropriate data model does not require assumptions about the residuals of the model (Derville et al., 2016).

Detection data were divided into a time‐series of 5‐min bins starting from 1st December 2019 and ending on 10th March 2020. The BRT was then constructed with a binomial response of present (1) or absent (0) within each 5‐min bin. The final time‐series contained 28,654 × 5‐min bins of presence and absence observations.

**FIGURE 3:**
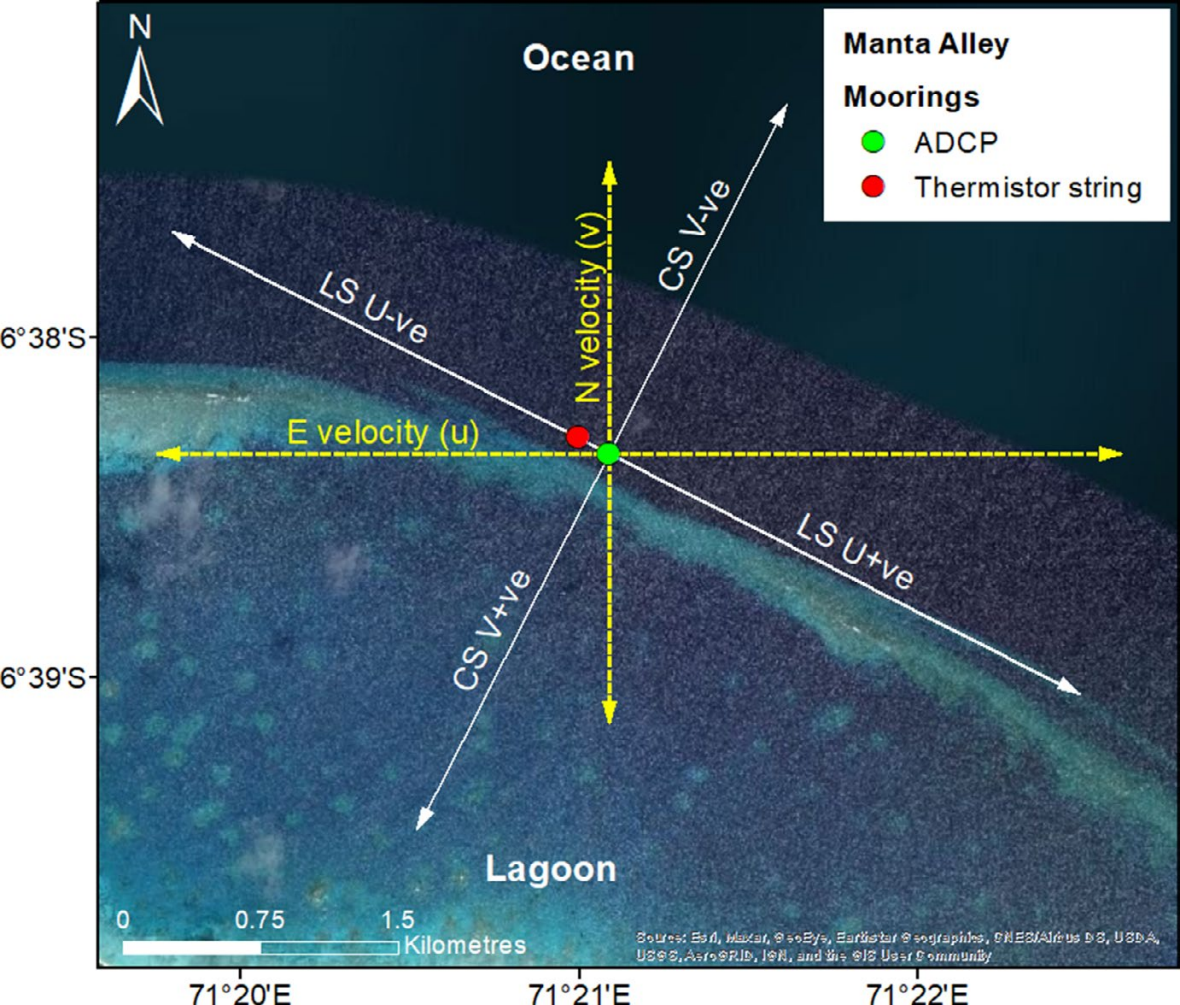
Original current u and v components (yellow dashed lines) and after clockwise rotation 117° relative to north (white lines). Arrows on the white lines show the direction of longshore u (LS U −ve and LS U +ve) and cross‐shore v (CS V −ve and CS V +ve). Showing mooring locations: long thermistor string (red dot) and subsurface ADCP moorings (yellow dot)

Nine predictor variables representing temperature (1–2), zooplankton biomass (3), ocean currents (4–8), and tide (9), all of which have been shown to influence *M. alfredi* occurrence (Anderson et al., 2011; Harris et al., 2020; O’Shea et al., 2010), were selected for inclusion (Table 1). Temperature variables included the following: temperature at 2 m above the seabed (temp 2 m) (1), and 50 m above the seabed (temp 50 m) (2), sampled every 5 s using RBR Concerto CTDs. Data were pooled into the same 5‐min bins as the presence and absence data. The mean temperature for each 5‐min bin was then calculated from the 60 data points. For zooplankton biomass (3), acoustic backscatter was used as a proxy. Data were taken from beam 1 of the Nortek Signature 500 kHz ADCP, aligned with *x* positive and a center frequency of 500 kHz with a bandwidth of 25 kHz and retrieved from the Nortek Average AD2CP file with a sample interval of 10 min. The instrument has a vertical resolution of 2 m per bin, and the first 25 bins were taken for data processing (0.5–50.5 m range from the instrument). The vertical profile at each time step was filtered with a running median window with a length of 3 and a maximum deviation of 1. These parameters were chosen based on observations in the data that any areas of amplified return (likely due to large targets which are not zooplankton) were constrained to the extent of a single bin due to the relatively large size of the 2 m bins when compared to the observed target size. A depth–mean value was then calculated for each time step and linearly interpolated into the 5‐min bins. Ocean current data (4) included vertical velocity, for which a depth–mean was calculated from the same average data and bin selection as backscatter, but with no filtering applied due to the low noise level in averaged velocity measurements. Data were then interpolated on to a 5‐min time scale. Ocean current data also included the eastward (u) component at 8.5 and 48.5 m above the seabed, and northward (v) component 8.5 and 48.5 m above the bed. Data were obtained from the Nortek Average AD2CP file, which provides a reading of component velocity at 10‐min intervals; each reading covers a time period of 120 s and is composed of 48 independent samples (0.4 Hz), giving an overall measurement uncertainty of <1 cm/s for horizontal velocity measurements. Ten‐min data were interpolated onto a 5‐min timescale using linear sampling. Each variable is representative of a single 2 m depth bin, chosen to be safely out of the influence range for ringing and sidelobe interference (5.5 and 45.5 m from the transducer, respectively). Both u and v velocity components were then rotated clockwise 117° relative to north, to align with the slope in Manta Alley (adjusted positive and negative directions, shown in Figure 3), resulting in the ocean current predictor variables: cross‐shore current at 48.5 m above the bed (CS current (v) 48.5 m) (5), cross‐shore current at 8.5 m above the bed (CS current (v) 8.5 m) (6), longshore current at 48.5 m above the bed (LS current (u) 48.5 m) (7), and longshore current at 8.5 m above the bed (LS current (u) 8.5 m) (8). To estimate tidal phase, pressure data were taken from the lower RBR Concerto CTD (depth 64.1 m) and converted to depth data using RSKTools inbuild conversion function. Data were cleaned with a median filter and averaged with a running window (both size 501 points). The Matlab inbuilt find peaks function was then used and ran twice to pick out both high and low tides by inverting the data on one run. A 5.5 hr minimum peak spacing was specified to further reduce susceptibility to noise, and the resulting data points were visually validated against the raw depth data. The variable time relative to high tide (9) was then calculated with high tide as zero and negative hours before (flood) and positive hours after (ebb; Peel, Stevens, et al., 2019).

**TABLE 1:** Description of the predictor variables used in boosted regression trees analysis of tagged *Mobula alfredi* occurrence at Manta Alley

Predictor no.	Predictor	Unit	Mean	Description
1	Temp 2 m	°C	27.7	Temperature 2 m above the bed (depth 64.1 m)
2	Temp 50 m	°C	29.1	Temperature 50 m above the bed (depth 13.4 m)
3	Backscatter	dB	46.1	Depth–mean linearly interpolated into 5‐min bins
4	Cross‐shore (v) 48.5 m	m/s	−0.007	Surface current 48.5 m above the bed (depth 17.6 m) flowing 27° (−ve) and 207° (+ve) relative to *N*
5	Cross‐shore (v) 8.5 m	m/s	−0.016	Near‐bed current 8.5 m above the bed (depth 57.6 m) flowing 27° (−ve) and 207° (+ve) relative to *N*
6	Longshore (u) 48.5 m	m/s	−0.095	Near‐surface current 48.5 m above the bed (depth 17.6 m) flowing 117° (−ve) and 297° (+ve) relative to *N*
7	Longshore (u) 8.5 m	m/s	−0.078	Near‐bed current 8.5 m above the bed (depth 57.6 m) flowing 117° (−ve) and 297° (+ve) relative to *N*
8	Vertical velocity	m/s	0.002	Upward (+ve) and downward (−ve) current flow
9	Time to high tide	0.083 hr	0.014	Time relative to high tide in steps of 5‐min (0.083h) with high tide zero, negative values before (flood) and positive values after (ebb)

Note

All predictors are in 5‐min means unless otherwise specified. All distances are meters above the seabed. Mean values show the value at which the predictor is held for partial dependency and interaction plots.

All models were fitted using the gbm.step() function of the dismo R package (Hijmans et al., 2017). Initial models were built to find suitable settings for four parameters: tree complexity (*tc*), which specifies the number of interactions that should be modeled, learning rate (*lr*), which regulates the contribution of each tree to the growing model, bag fraction (*bf*), which controls stochasticity by randomly selecting (without replacement) a specified subset of the data at each iteration and step size (*ss*), which controls the number of trees which should be added at each iteration (Elith et al., 2008). The following parameter settings were tested: *tc* = 1–6, *lr* = 0.01, 0.005, 0.001 and 0.0001, *bf* = 0.5, 0.7, 0.9, *ss* = 25 and 50, resulting in 144 models.

Ten‐fold cross‐validation (CV) was applied to assess model performance, whereby the model is fitted to training data and then is tested against a withheld portion (hold‐out sample) of the dataset (Elith et al., 2008). The model's ability to fit the withheld data was then measured by comparing the area under the receiver operating characteristic curve (AUC) test statistic (Dedman et al., 2017; Froeschke et al., 2010) for both the training data (_T_AUC) and hold‐out sample (cross‐validation AUC, _CV_AUC; Dedman et al., 2017; Elith & Leathwick, 2017). The AUC classification ranges from 0 to 1, whereby: <0.5 (fail), 0.6–0.7 (poor), 0.7–0.8 (acceptable), 0.8–0.9 (excellent), >0.9 (outstanding; Hosmer & Lemeshow, 2000). The difference between the _T_AUC and the _CV_AUC (ΔAUC) indicates the level of overfitting of the primary sample (Dedman et al., 2017). Therefore, better model performance is categorized by higher AUC values for both _T_AUC and _CV_AUC, but a lower ΔAUC (Dedman et al., 2017).

The percentage of deviance explained by the model was determined using the pseudo determination coefficient (*D* ^2^), calculated using the following form (Nieto & Mélin, 2017):

$$D^{2}=1‐\left(\text{residual}\;\text{deviance/total}\;\text{deviance}\right).$$

The final model was fitted with *tc* = 6, *lr* = 0.005, *bf* = 0.7, and *ss* = 50 (Table S2). The relative contribution of predictor variables to the BRT model is measured by averaging the number of times a variable is chosen for splitting and the squared improvement resulting from these splits (scaled to 100 across all the variables; Elith et al., 2008). To ensure noninformative predictors were not hindering model performance, pairwise correlation coefficients and variance inflation factor (VIF) estimates (Jouffray et al., 2019) were calculated, all were in an acceptable range; coefficients <0.6 and/or VIF estimates <3.5 (Jouffray et al., 2019; Table S1; Figure S1).

Due to the complex nature of BRTs, model results cannot be easily visualized. Therefore, partial dependency and interaction plots were generated for interpretation. The plots display the results of the predicted effect on tag detection probability for a given predictor, or pair of predictors, after accounting for the mean effects of all other predictors (Elith et al., 2008; Hastie et al., 2009). Confidence intervals (95%) for the partial dependency plots were obtained from 1,000 bootstrap replicates (Jouffray et al., 2019). For interaction plots, 100 bootstrap resampling was used to test the significance of the strongest interactions (Jouffray et al., 2019; Pinsky & Byler, 2015; interaction strength >100) by randomly sampling the occurrence of *M. alfredi* at each location before re‐fitting the BRT models (Jouffray et al., 2019). The size of the interaction was then used to generate a distribution under the null hypothesis of no interaction among predictors (Jouffray et al., 2019).

## 3 RESULTS

### 3.1 Detection and resident event summary

Acoustic transmitter tags were deployed on eleven female (adults = 5, sub‐adults = 4, juvenile = 2) and nine male (adults = 3, juvenile = 6) *M. alfredi* (Table 2). Nineteen of the 20 tags returned useable tracks (Table 2). No detections have been recorded for three of the 19 tags since November 21, 2019 (CG‐MA‐0120), December 27, 2019 (CG‐MA‐0161), and January 20, 2020 (CG‐MA‐0141). However, it is not possible to distinguish between acoustic tag loss and emigration from the study area, and relatively long temporal gaps between detections of *M. alfredi* in the Chagos Archipelago have previously been reported (Andrzejaczek et al., 2020). Therefore, the current status of these tags has been recorded as “unknown” rather than “inactive,” pending further data collection.

**TABLE 2:** Summary of *Mobula alfredi* acoustic tag deployments (*n* = 19), tracking, detections, and Residency Index (RI) for Egmont Atoll

Manta ID	Sex	Maturity status	Tag ID	Deployment date	Deployment location	Lat	Long	Last detection	Total No. of detection	Tracking days	Detection days	Residency Index (RI%)	Status
CG‐MA‐0035	F	Sub‐adult	890	20/11/2019	Ile Sipaille	−6.67	71.32	13/03/2020	1,159	115	92	80	ACTIVE
CG‐MA‐0046	F	Adult	892	25/11/2019	Ile Tattamucca	−6.69	71.38	10/03/2020	846	107	61	57	ACTIVE
CG‐MA‐0070	F	Adult	891	01/12/2019	Ile Lubine	−6.67	71.32	17/03/2020	1,059	108	46	43	ACTIVE
CG‐MA‐0088	M	Juvenile	897	28/11/2019	Ile Lubine	−6.67	71.32	15/03/2020	907	109	43	40	ACTIVE
CG‐MA‐0094	F	Sub‐adult	894	30/11/2019	Ile Sipaillle	−6.66	71.31	13/03/2020	1,116	105	67	64	ACTIVE
CG‐MA‐0112	M	Juvenile	893	01/12/2019	Ile Lubine/Sipaillle	−6.66	71.31	13/03/2020	1,129	104	49	47	ACTIVE
CG‐MA‐0117	F	Sub‐adult	887	20/11/2019	Ile Lubine	−6.67	71.32	13/03/2020	718	115	50	44	ACTIVE
CG‐MA‐0118	M	Juvenile	900	19/11/2019	Ile Sipalle	−6.66	71.31	11/03/2020	678	114	41	36	ACTIVE
CG‐MA‐0119	F	Juvenile	901	19/11/2019	Ile Sipalle	−6.67	71.32	13/03/2020	801	116	54	47	ACTIVE
CG‐MA‐0120	F	Adult	902	19/11/2019	Ile Sipalle	−6.67	71.32	21/11/2019	15	3	2	76	UNKNOWN
CG‐MA‐0121	M	Juvenile	903	19/11/2019	Ile Sipalle	−6.67	71.32	13/03/2020	1,770	116	84	73	ACTIVE
CG‐MA‐0124	M	Adult	885	20/11/2019	Ile Lubine	−6.67	71.32	12/03/2020	559	114	44	39	ACTIVE
CG‐MA‐0125	M	Juvenile	895	20/11/2019	North IDR Cleaning St	−6.64	71.32	14/03/2020	1,272	116	60	52	ACTIVE
CG‐MA‐0139	F	Adult	899	25/11/2019	Ile Tattamucca	−6.69	71.38	15/03/2020	763	112	73	66	ACTIVE
CG‐MA‐0140	M	Juvenile	898	30/11/2019	North IDR Cleaning St	−6.64	71.32	13/03/2020	711	105	25	24	ACTIVE
CG‐MA‐0141	F	Adult	889	28/11/2019	Ile Lubine	−6.67	71.32	20/01/2020	411	54	31	58	UNKNOWN
CG‐MA‐0142	F	Sub‐adult	884	02/12/2019	Ile Tattamucca	−6.70	71.40	15/03/2020	1,216	105	59	56	ACTIVE
CG‐MA‐0151	M	Adult	896	01/12/2019	Ile Lubine	−6.68	71.33	12/03/2020	794	103	62	60	ACTIVE
CG‐MA‐0161	M	Adult	888	02/12/2019	Ile Carre Pate	−6.68	71.35	27/12/2019	41	26	7	27	UNKNOWN

Note

Status refers to the known status of tags at the time of last detection data download (17th March 2020).

**FIGURE 4:**
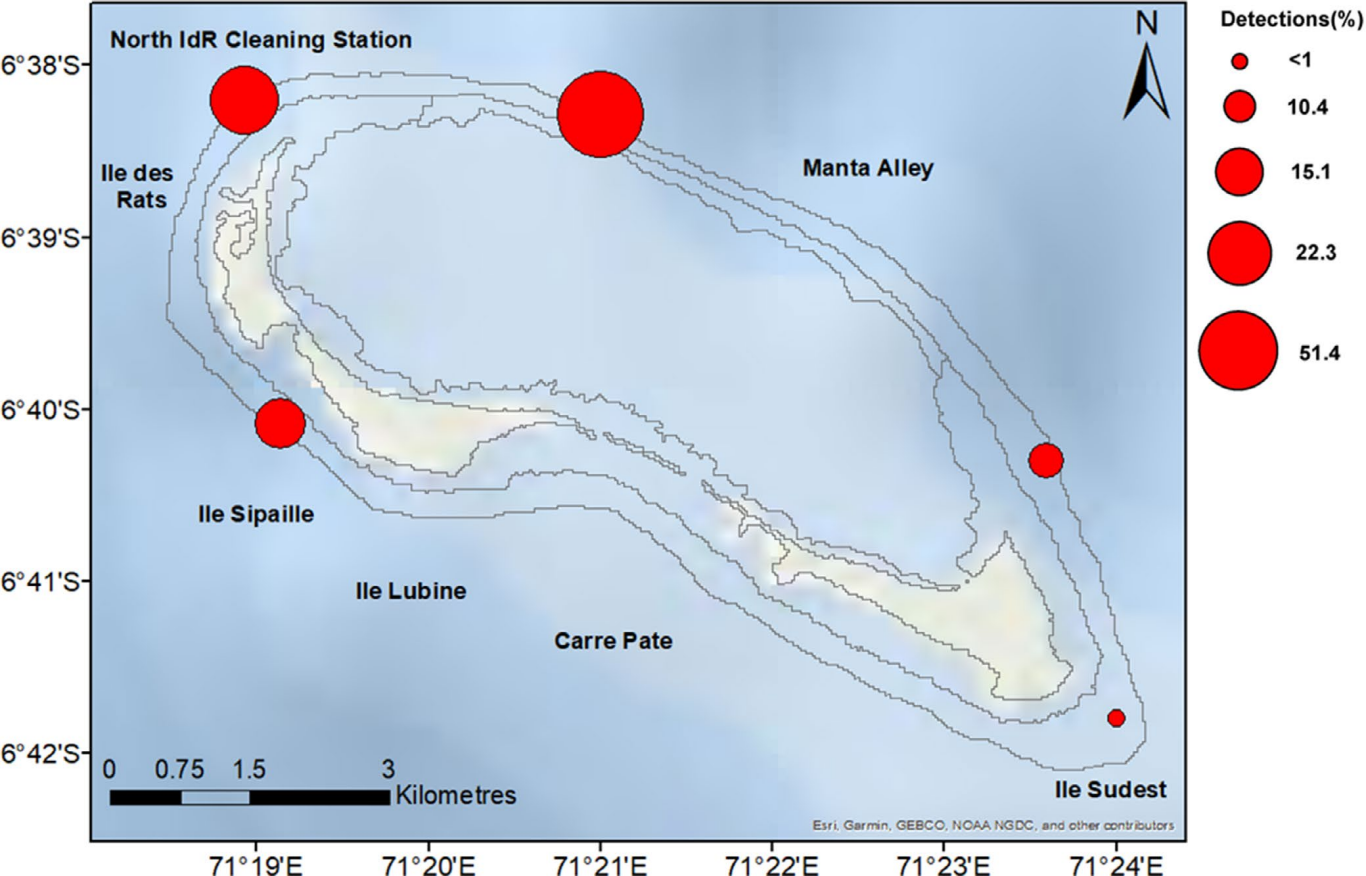
Percentage of detections at each site

There were a total of 15,965 detections during the study period (Table 2). The highest percentage of detections occurred at the acoustic receiver deployed on the oceanographic mooring in Manta Alley (51.4%), followed by North IdR Cleaning Station (22.3%; Figure 4).

The overall distribution of detections by hour of the day shows 70.9% of detections occurred at Egmont Atoll during the day (06:00–18:00; Figure 5). For adults, only 18.3% of detections occurred at night (19:00–05:00), while 35% of detections occurred at night for juveniles.

**FIGURE 5:**
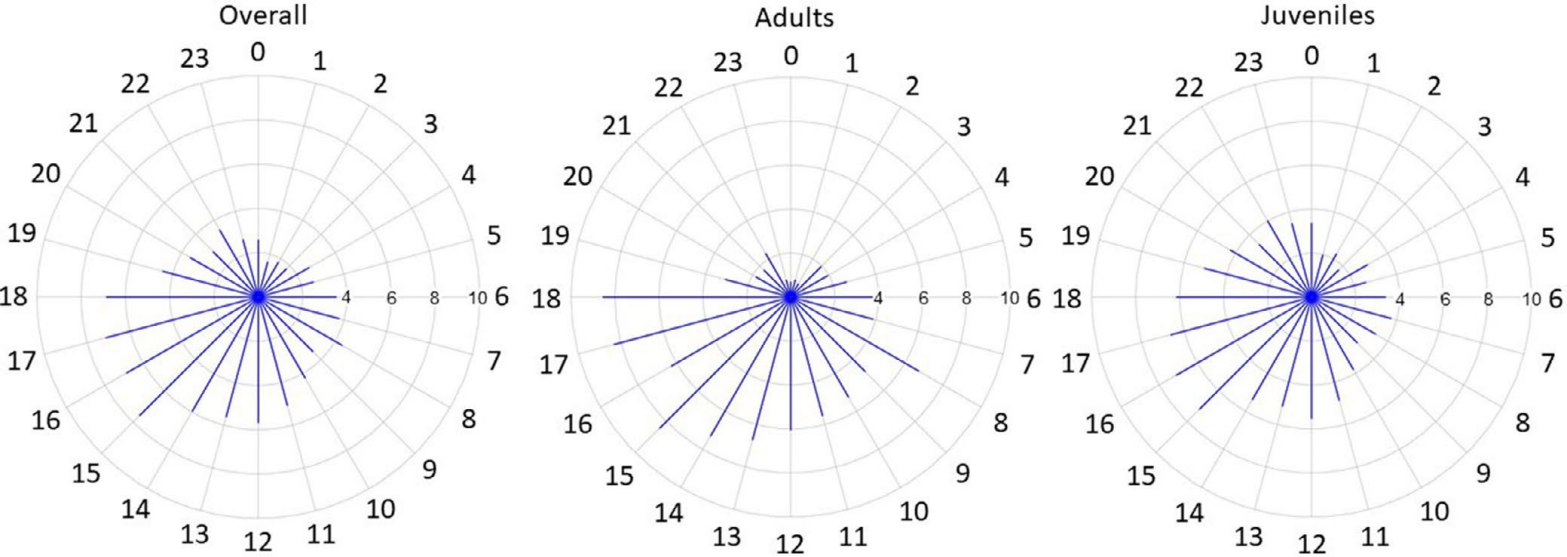
Percentage distribution of detections by hour of the day at Egmont Atoll for all tagged *M. alfredi* (left), adults only (middle), and juveniles only (right)

The mean total time between tag deployment when the tags first began transmitting until the end of the study, when the detection data were downloaded, was 113 ± 5 day (range 106–119 days). During this time, tagged *M. alfredi* were tracked (first to last tag detection) for a mean of 97 ± 32 days (range 3–116 days), with a mean of 50 ± 23 detection days (range 2–92 days). Residency indices show that tagged *M. alfredi* were detected at Egmont Atoll for a mean of 52% of the days they were tracked (RI = 52 ± 15.7%), with a minimum and maximum RI of 24% and 80.3%, respectively (Table 2). Mean residency indices were similar for both adults and juveniles (including sub‐adults), which were 53 ± 16% and 51 ± 16%, respectively.

**FIGURE 6:**
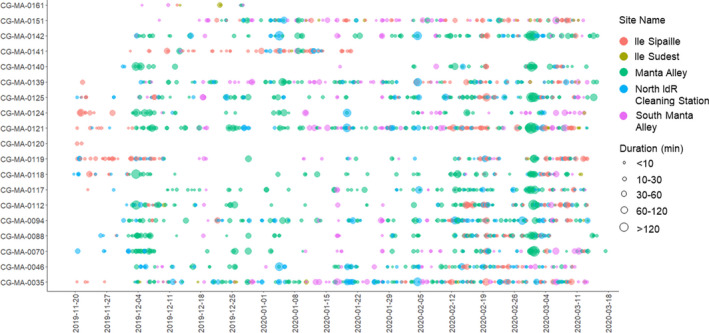
Resident events at each site showing location (by color) and time at the location (by size)

Overall, 2074 resident events were recorded for 19 M*. alfredi* (Figure 6). The highest number of resident events occurred at Manta Alley (837), totaling 22,188 min (369.8 hr). Manta Alley also had the longest individual mean resident event time (27 ± 51; Table 3), with the longest resident event of 489 min (8.2 hr) by a juvenile male (manta‐ID CG‐MA‐0125). Of the 837 resident events, a total of 35 lasted >120 min, of which 11 were female (adult = 5, juvenile = 6) and 24 were males (adult = 3, juvenile = 21).

**TABLE 3:** Summary of acoustic receiver deployment locations, recording times, and resident event durations

Location	Deployed	Lat	Long	Depth (m)	Height above seabed (m)	No. days recording	No. *M. alfredi*	Mean resident event (min ± *SD*)	Max resident event (min)
Ile Sipaille	19/11/2019	71.32	−6.67	14.6	1.5	116	19	15 ± 26	200
Ile Sudest	01/12/2019	71.40	−6.70	15	1.8	104	11	5 ± 10	56
Manta Alley	30/11/2019	71.35	−6.64	70	48	109	18	27 ± 51	489
North IdR Cleaning Station	19/11/2019	71.32	−6.64	13.6	1.6	116	17	19 ± 36	294
South Manta Alley	30/11/2019	71.39	−6.67	14.2	1.8	105	18	16 ± 26	133

The lowest number of residency events occurred at Ile Sudest (41), totaling 191 min (3.1 hr). Mean resident event time was 5 ± 10 min, with a maximum resident event time of 56 min by an adult male (manta‐ID CG‐MA‐0161).

### 3.2 Environmental influences: boosted regression trees

Model performance evaluation for the BRT, including all nine predictors, had outstanding and excellent predictive performance for the training (_T_AUC = 1) and cross‐validated (_CV_AUC = 0.89) data, respectively, with minimal evidence of overfitting (ΔAUC = 0.11). The estimated *D* ^2^ suggests that 72% of the deviance was explained (Table S2).

**FIGURE 7:**
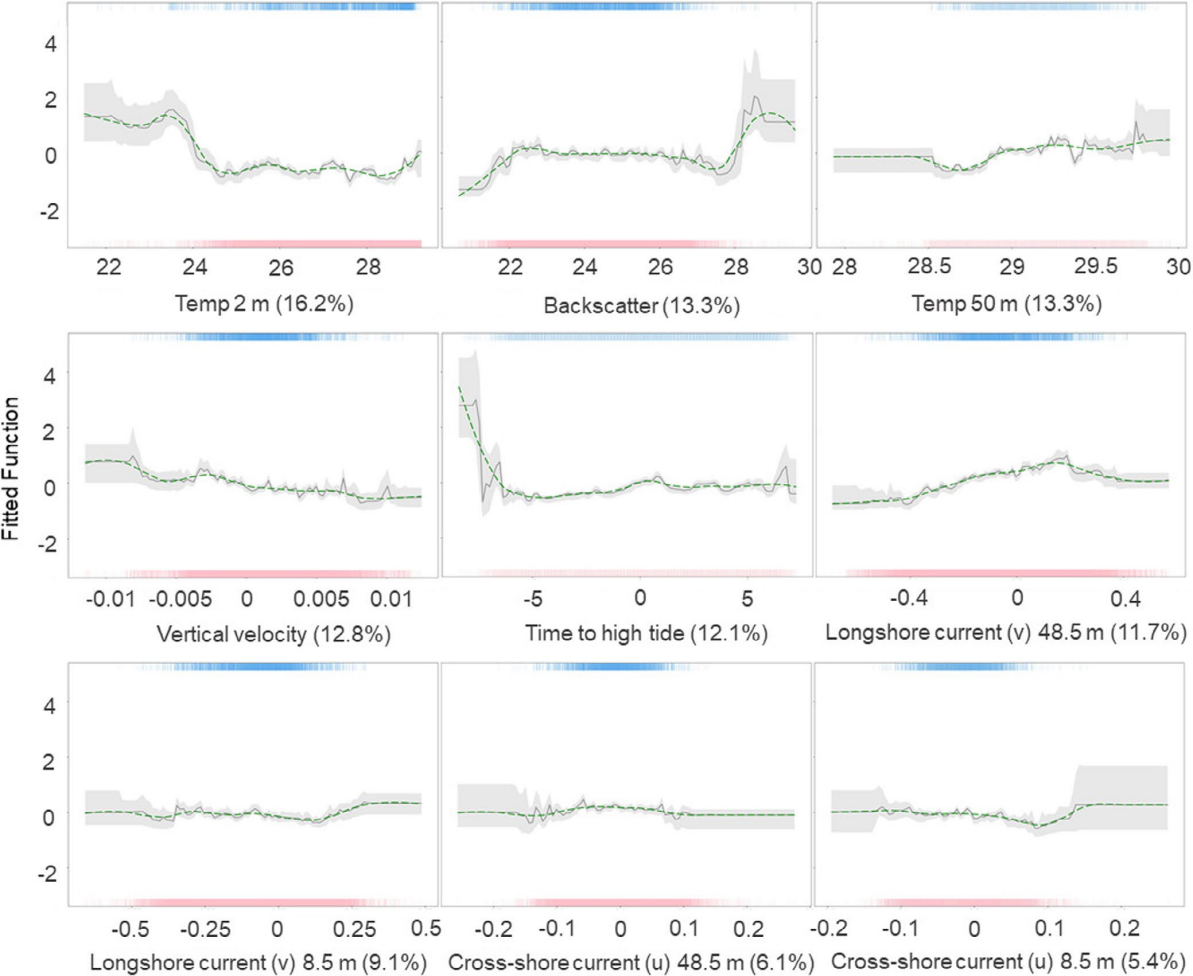
Partial dependency plots showing the effect of each predictor variable: temperature 2 m above the bed (Temp 2 m), depth–mean backscatter intensity linearly (Backscatter), temperature 50 m above the bed (Temp 50 m), upward (+ve) and downward (−ve ) current flow (Vertical velocity), time relative to high tide in steps of 5‐min (0.083 hr) with high tide zero, negative values before (flood) and positive values after (ebb) (Time to high tide), near‐surface current 48.5 m above the bed (depth 17.6 m) flowing 117° (−ve ) and 297° (+ve) relative to *N* (Longshore (u) 48.5 m), near‐bed current 8.5 m above the bed (depth 57.6 m) flowing 117° (−ve ) and 297° (+ve) relative to *N* (Longshore (u) 8.5 m), near‐surface current 48.5 m above the bed (depth 17.6 m) flowing 27° (−ve ) and 207° (+ve) relative to *N* (Cross‐shore (v) 48.5 m), near‐bed current 8.5 m above the bed (depth 57.6 m) flowing 27° (−ve ) and 207° (+ve) relative to *N* (Cross‐shore (v) 8.5 m), on the occurrence of tagged *M. alfredi* at Manta Alley while keeping all other variables at their mean. The green line shows smoothed partial decency. Rugs display the distribution of the data for presence (top, blue), and absence (red, bottom)

Partial dependency plots (Figure 7) indicate that the probability of detections decreased with increased near‐bed temperature (temp 2 m, 16.2%), and increased with increased backscatter strength (13.3%) and near‐surface temperature (temp 50 m, 13.3%). Detection probability was higher with greater downward vertical velocity (12.8%), and during the early stages of a flood tide (12.1%), approximately two hours following low tide when near‐surface longshore current velocity (longshore (v) 48.5 m, 11.7%) was approximately 0.2 m/s near the surface and 0.3 m/s near the seabed (longshore (v) 8.5 m, 9.1%). For cross‐shore currents, detection probability increased when near‐surface currents were flowing offshore (cross‐shore (u) 48.5 m, 6.1%) at approximately −0.05 m/s, and when near‐bed currents (cross‐shore (u) 8.5 m, 5.4%) were flowing inshore at approximately 0.15 m/s.

Eight significant pairwise interactions occurred between eight predictors (Table 4; Figure S2). These interactions should not be considered in isolation, as they may arise separately or simultaneously, and may be affected by other variables. However, they provide insight into the estimated influence of several paired‐environmental processes which can increase the probability of tag detections. For example, tag detections probability was highest when upward currents speed (vertical velocity) was increased, and the near‐surface temperature (Figure S2a) was warmer (temp 50 > 29.5°C). Tag detection probability also increased with cooler near‐bed temperature (temp 2 m < 24°C) and increased near‐surface temperatures (temp 50 m > 29.5°C; Figure S2b). It also increased when near‐bed cross‐shore currents (cross‐shore (u) 8.5 m) were of a high +ve velocity (>0.15 m/s; flowing into the lagoon) and backscatter was >55 (Figure S2c), and with high temp 2 m (>29°C) and backscatter >55 (Figure S2d).

**TABLE 4:** Pairwise interactions between predictor variables with all other variables held to their respective mean (Table 1)

Plot	Predictor 1	Predictor 2	Interaction size	Nature of interaction increasing tag detection probability	Max detection probability (%)
S2a	Temp 50 m	Vertical velocity	768.77	Warmer near‐surface temperature and increased upward vertical velocity	90
S2b	Temp 50 m	Temp 2 m	741.86	Cooler near‐bed temperature and increased near‐surface temperature indicating the water column are stratified. Warmer near‐bed temperature (>28.5°C) with similar near‐surface temperature (28–28.5°C)	92
S2c	Cross‐shore (v) 8.5 m	Backscatter	427.88	Near‐bed longshore currents flowing at velocity of >0.15 m/s (+ve, flowing into the lagoon) and high backscatter intensity (>55)	85
S2d	Temp 2 m	Backscatter	405.69	Warmer near‐bed temperature (>29.5°C) and high backscatter intensity (>55)	98
S2e	Longshore (u) 8.5 m	Time to high tide	165.86	During the early stages of flood with increasing near‐bed longshore current velocity (+ve, flowing from North IdR Cleaning Station toward South Manta Alley) flowing from the	86
S2f	Longshore (u) 48.5 m	Time to high tide	124.66	During the early stages of flood with moderate velocity (approximately 0.3–0.4 m/s) longshore near‐surface current (+ve, flowing from North IdR Cleaning Station toward South Manta Alley)	66
S2g	Temp 50 m (53%)	Time to high tide	182.1	During the early stages of flood with cooler near‐surface temperature (<29°C) and during ebb close to low tide with warmer near‐surface temperature (>29.5°C)	59
S2h	Longshore (v) 8.5 m	Backscatter	100.63	Longshore near‐bed currents flowing at moderate to the maximum speed observed (−0.66 m/s) from South Manta Alley toward North IdR Cleaning Station, and high backscatter intensity (>55)	73

Note

Higher interaction size values indicate a more substantial interaction effect; near zero indicates negligible interactions. All interactions were significant (*p* < .01). The suggested influence of the interaction on the probability of detections is described along with the maximum detection probability estimated for each interaction.

## 4 DISCUSSION

Overall, *M. alfredi* residency at Egmont Atoll, measured by the residency index (RI), was high (mean RI = 52%), which supports previous reports that Egmont Atoll provides key habitats for this species (Andrzejaczek et al., 2020; Harris, 2019). Similar high levels of residency have been observed in the Red Sea (mean RI = 65%; Braun et al., 2015) and at the Amirante Islands of Seychelles (mean RI = 62%; Peel, Stevens, et al., 2019). Adult and juvenile *M. alfredi* displayed similar residency at Egmont Atoll, which is in contrast to patterns observed in Seychelles, where the RI was lower for adults (Peel, Stevens, et al., 2019), indicating that the *M. alfredi* habitat at Egmont Atoll is perhaps consistently important for all life stages. Alternatively, the similar residency of adults and juveniles could be attributed to the acoustic array design (Peel, Stevens, et al., 2019). To establish a more robust RI, future research would benefit from increased spatial coverage, including the deployment of acoustic receivers in locations which may be frequented by juveniles such as inside the lagoon.

Overall, detection data display diel behavior patterns, with the highest percentage of detections at Egmont Atoll occurring during the day. Similar diel patterns have been recorded during studies of various *M. alfredi* subpopulations, where individuals frequented shallow coastal and island reef systems more often during daylight hours (Couturier et al., 2018; Dewar et al., 2008; Jaine et al., 2012; Peel, Stevens, et al., 2019; Setyawan et al., 2018). These patterns may be associated with the species use of cleaning stations, where cleaner fish are only active during the day (Côté, 2000). Diel movement patterns may also be associated with efficient foraging strategies. For example, *M. alfredi* may predominately frequent shallow reef habitats during the day to feed on reef‐associated zooplankton which can accumulate in surface waters over shallow reefs when avoiding predation from reef‐dwelling diurnal consumers (Alldredge & King, 2009; Leichter et al., 2013). At night, *M. alfredi* may then travel offshore to forage (Couturier et al., 2018; Dewar et al., 2008; Jaine et al., 2012) when diel vertically migrating zooplankton ascends into warmer water (Braun et al., 2014; Couturier et al., 2018; Dewar et al., 2008). This hypothesis is supported by stable isotope analysis, which indicates that a large proportion of *M. alfredi* diet is made up of both near‐surface and demersal zooplankton (Couturier et al., 2013; Peel, Daly, et al., 2019). The diel *M. alfredi* movement pattern was less pronounced for juveniles, which were more frequently detected at night than adults, suggesting that juvenile *M*. *alfredi* remain in shallower reef habitats longer. This pattern has also been observed in other subpopulations and is likely a predator avoidance strategy by the more vulnerable juveniles (Cerutti‐Pereyra et al., 2014; Peel, Stevens, et al., 2019; Stewart et al., 2018). Their smaller body size may also make it less energetically efficient for juveniles to travel offshore (Nøttestad et al., 1999; Peel, Stevens, et al., 2019), and/or their foraging experience may be limited (Peel, Stevens, et al., 2019).

Manta Alley had the highest number of detections, and there were repeated resident events for 18 of the 19 tagged individuals, indicating a high level of site fidelity. Site fidelity is a well‐reported characteristic of *M*. *alfredi*, having been observed in photographic identification, acoustic telemetry, and satellite tagging studies (Couturier et al., 2018; Deakos, 2011; Dewar et al., 2008; Jaine et al., 2014; Kessel et al., 2017; McCauley et al., 2014; Stevens, 2016). Site fidelity has been attributed in part to the species’ reliance on specific habitats, which provide a sufficient food resource, protection from predation, and opportunities to clean, socialize, and reproduce (Jaine et al., 2014; McCauley et al., 2014; Perryman et al., 2019; Stevens, 2016). Resident events were longer at Manta Alley than at any other location. The depth of the majority of the area within the range of the acoustic receiver is greater than 40 m. As 40 m is the maximum depth of occurrence for cleaner fish in the Chagos Archipelago (Kuiter, 2014), it is unlikely that these extended resident events at Manta Alley are associated with cleaning activities. Furthermore, in‐water observations at this site by the authors during this study observed large *M. alfredi* aggregations feeding (~40 individuals) on the surface down to 20 m on several occasions, while no cleaning stations were identified. Therefore, the most likely driver of the visitation patterns recorded for *M. alfredi* at this site during this study is foraging opportunities. For *M. alfredi* foraging activities to be energetically efficient, high densities of prey are required (Armstrong et al., 2016). The BRT model suggests that *M. alfredi* presence at Manta Alley is associated with various fine‐scale oceanographic processes, which could be combining to enhance localized zooplankton abundance.

A high tag detection probability of *M. alfredi* occurred with cold near‐bed and warm near‐surface temperature, and probability increased with increasing difference between these temperatures. Extreme short‐term fluctuations in near‐bed temperatures may be associated with the intrusion of cold water created by internal waves which disrupt the thermocline (Shanks et al., 2014). Enhanced concentrations of zooplankton often occur at the thermocline, the thickness of which can be increased by internal waves (McManus et al., 2005). These internal waves break as they interact with the steep slope of an atoll leading to the formation of cold‐water bores which propagate up the slope (Hosegood et al., 2019; Woodson, 2018). Bores enhance the upward transport of organisms, and thus the concentration in surface waters (Stevick et al., 2008), which may provide efficient foraging opportunities for the zooplanktivorous *M. alfredi*. The upward propagation of cold‐water bores has been observed to vary tidally (Hosegood et al., 2019; Leichter et al., 1996), and can become more frequent during a flood tide leading to a pulsed delivery of organisms (Leichter et al., 1996; Woodson, 2018). Here, tag detection probability was high during the early stages of a flood tide and was also increased by the interaction effect between a flood tide and cooler near‐surface temperature, which may indicate that cold‐water bores propagate up the slope (Leichter et al., 1996). Plankton sampling and oceanographic measurements obtained inside the lagoon also indicate that increased zooplankton abundance is associated with the transfer of plankton into the lagoon from the intrusion of cold‐water bores created by breaking internal waves (Sheehan et al., 2019). The intrusion of cold water may also provide metabolically advantageous feeding conditions for *M. alfredi* by reducing the energetic cost of feeding activities (Lawson et al., 2019).

In the current study, tag detection probability also increased with the interaction between high‐velocity near‐bed cross‐shore currents flowing inshore and high levels of backscatter. This interaction may indicate that zooplankton is being carried from the thermocline into the lagoon during a flood tide and is likely pumped back out during ebb. Due to the partially enclosed morphology of the lagoon, water entering is likely to be restricted by the narrow subtidal passages. Even with a low tidal amplitude, strong jet‐like currents can be generated (Dumas et al., 2012), which may increase the density of inflowing (outflowing) zooplankton approaching low tide (in the early stages of flood), as suggested by the in‐water observations of the current study. During these events, mobile zooplankton may actively seek refuge zones to avoid predation or import into (export from) the lagoon (Pagano et al., 2017). Refuge zones include the thermocline and behind shallow back reefs (Leichter et al., 2013), where zooplankton become concentrated further, providing dense assemblages of prey for *M. alfredi*. Similar theories of zooplankton retention, which are also related to tide phase, have been suggested in other regions (Armstrong et al., 2016; Stevens, 2016).

The BRT also provided some evidence of the presence of Langmuir Circulation (LC), which can trap and concentrate particles in the water column (Smith, 2001). The process is driven by wind and waves which produce helical vortices that appear as rotating cells that rotate perpendicular to the wind direction (Smith, 2001). The interaction effect between high‐velocity near‐bed longshore currents flowing, when near‐surface currents were flowing in the opposite direction, and high backscatter intensity could be evidence of LC cells. Alternating cells rotate in opposite directions leading to areas of convergence and divergence (Talley et al., 2011). Downwelling, which increased the probability of tag detections, occurs in areas of convergence where plankton, other organisms, and particles become trapped in highly concentrated bands (Kingsford et al., 1991; Thorpe, 2004). These bands may provide ideal foraging opportunities for *M. alfredi*. As LC can persist for hours or even days (Gargett et al., 2004), it could potentially be associated with resident events which last longer than the influence of the tide (>2 hr). The characteristic surface “slicks” which often accompany LC have also been regularly observed by authors in Manta Alley, further supporting this suggestion.

Under well‐mixed conditions, LC can develop “super‐cells” which extend the full depth of the water column (Gargett et al., 2004). These super‐cells can transport organisms and partials from depths up into the water column where they become concentrated in the narrow bands of the convergence zones (Gargett et al., 2004; Kukulka et al., 2012). Potential evidence of the presence of super‐cells and their positive influence on *M. alfredi* detection probability is apparent with the interaction effects between high near‐bed temperature near‐bed temperature (>28.5°C) and lower near‐surface temperature (28–28.5°C), indicating a well‐mixed water column, warmer near‐bed temperature (>28.5°C), and high backscatter, and increased downward vertical velocity (downwelling) and low near‐surface temperatures (<28.5°C). However, high backscatter could also be caused by sediment resuspension events which can be induced by LC super‐cells (Gargett et al., 2004). There may be evidence of the effect of LC on *M. alfredi* visitation and behavior patterns in other regions. For example, in Komodo Marine Park in Indonesia, *M. alfredi* were observed feeding where there were surface slicks and a high density of particles in the water column (Dewar et al., 2008), which is characteristic of LC convergence zones (Kingsford et al., 1991). Around Lady Elliot Island (LEI) in Australia, sightings of foraging individuals and increased acoustic tag detection were correlated with wind speed (Couturier et al., 2018; Jaine et al., 2012). At LEI, *M. alfredi* sightings and tag detections peaked at wind speeds around 18 km/hr (approximately 5 m/s), an optimal speed for the development of LC (Langmuir, 1938; Plueddemann et al., 1996). At LEI, sightings were also associated with cooler sea surface temperatures, with a decrease in sightings and detections with increased temperature (Couturier et al., 2018; Jaine et al., 2012). Strong surface warming can lead to a breakdown of LC by disrupting the balance between wave‐forcing and thermal convection (density‐driven circulation; Li & Garrett, 1994; Min & Noh, 2004), which may reduce the density of prey, leading to a lower number of sightings and tag detections of *M. alfredi*.

There were some limitations to the current study. For example, the position of tagged *M. alfredi* in the water column and the distance the individual was from the acoustic receiver and oceanographic equipment could not be established. The acoustic doppler current profiler (ADCP) was also deployed on the edge of the range of the acoustic receiver. Therefore, it is possible that some of the changes in the oceanographic conditions which influence *M. alfredi* visitation patterns were not fully resolved here. These limitations may be mitigated in future by using acoustic transmitters which also deliver distance and depth information when the individual is detected. A reconfiguration of the mooring to include an ADCP in line with all other sensors would also be beneficial. Future research would also benefit from oceanographic monitoring within the lagoon passage and the Manta Alley feeding location concurrently to help further resolve the fine‐scale processes occurring at the lagoon‐ocean interface. Further investigation into the potential presence of LC and its influence on *M. alfredi* visitation patterns at Manta Alley is also required. Research should incorporate in situ wind directions measurements of the same spatial and temporal resolution as the longshore and cross‐shore current directions. These measurements should be accompanied by ADCP backscatter data to detect zones of high echo intensity and periods of downwelling in relation to *M. alfredi* visitation patterns, and plankton sampling to assess the content of the water column during LC events.

Studying *M. alfredi* at Egmont Atoll provides valuable insight into how the species respond to fine‐scale changes in their oceanographic environment, thus improving our current knowledge of *M. alfredi* foraging ecology. Evidence provided in this study suggests that the species regularly take advantage of feeding opportunities which are influenced by fine‐scale oceanographic processes that occur close to the lagoon‐ocean interface. These feeding opportunities appear to occur with tidal periodicity. During a flood tide, cold‐water bores frequently propagate up the slope, transporting zooplankton from the thermocline into the lagoon through the narrow inlet. During an ebb tide, the zooplankton then flows back out of the lagoon with the highest concentrations occurring close to low tide. Mobile zooplankton may become trapped and concentrated around reef structures as they attempt to avoid predation by moving back into deeper water. High concentrations of zooplankton which occur during these tidal phases are likely to be short‐lived, occurring in pulses, providing temporally limited feeding opportunities for *M. alfredi*. However, under suitable conditions, for example, in the presence of LC, highly concentrated bands of zooplankton may persist for hours, potentially providing extended feeding opportunities which result in *M. alfredi* spending long periods of time at the location.

## CONFLICT OF INTEREST

There are no competing financial, professional, or personal interests that might have influenced the performance or presentation of the work described in this manuscript. All authors have no conflict of interest to declare.

## AUTHOR CONTRIBUTIONS

**Joanna L Harris:** Conceptualization (lead); Data curation (lead); Formal analysis (lead); Investigation (lead); Methodology (lead); Writing‐original draft (lead); Writing‐review & editing (lead). **Phil Hosegood:** Conceptualization (supporting); Funding acquisition (equal); Investigation (supporting); Project administration (lead); Supervision (equal); Writing‐review & editing (supporting). **Edward Robinson:** Data curation (supporting); Investigation (supporting); Software (supporting); Writing‐review & editing (supporting). **Clare B Embling:** Supervision (equal); Writing‐review & editing (supporting). **Simon Hilbourne:** Investigation (supporting); Writing‐review & editing (supporting). **Guy M. W Stevens:** Conceptualization (supporting); Funding acquisition (equal); Supervision (equal); Writing‐original draft (supporting); Writing‐review & editing (supporting).

## Supporting information

Supplementary Material

## Data Availability

The data that support these findings are available from FigShare (https://doi.org/10.6084/m9.figshare.13139309) following a one‐year embargo from the date of publication to allow for further publication of research findings.
