# Supplementary material for: Fine‐scale oceanographic drivers of reef manta ray (Mobula alfredi) visitation patterns at a feeding aggregation site
Source: Ecol Evol. 2021 Mar 24;11(9):4588–604. doi: 10.1002/ece3.7357 (PMC8093739; doi:10.1002/ece3.7357)
Supplement: Supplementary file 1 — Supplementary Material [file ECE3-11-4588-s001.docx]

**Supplementary material**

**The tide waits for no manta: Fine-scale oceanographic drivers of reef manta ray, *Mobula alfredi*, visitation patterns at a feeding aggregation site**

**Table S1:** VIF scores for predictor variables

| **Predictor variable** | **VIF** |
| --- | --- |
| Temp 2 m | 1.070522994 |
| Temp 50 m | 1.008899318 |
| Backscatter | 1.136194438 |
| Longshore (v) 48.5 m | 2.394885481 |
| Longshore (v) 8.5 m | 3.186781946 |
| Cross-shore (u) 48.5 m | 1.590182677 |
| Cross-shore (u) 8.5 m | 1.847656189 |
| Vertical velocity | 1.061785125 |
| Time to high tide | 1.082522148 |

**
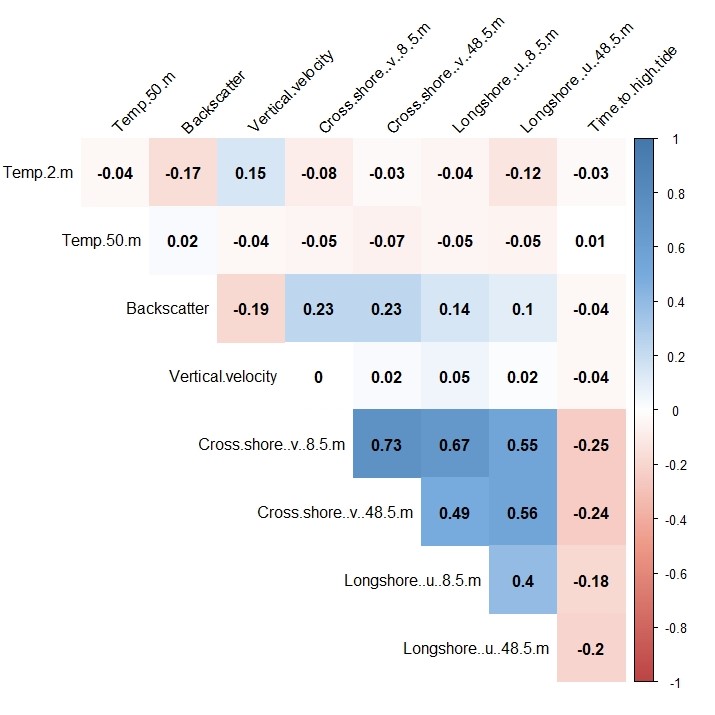
**

**Figure S1-** Correlation matrix for all predictors used in the boosted regression trees model.


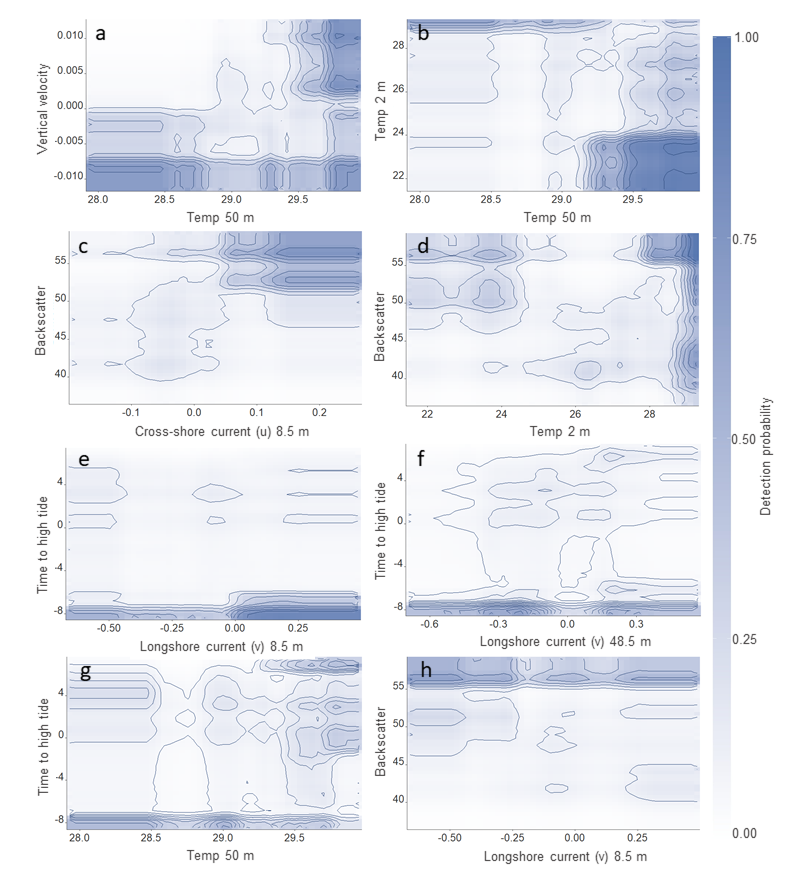


**Figure S2**: Pairwise interactions between predictor variables contour lines highlight the probability of *M. alfredi* tag detection at Manta Alley while keeping all other variables at their mean. All interactions were significant (*p*<0.01). Plot number related to the interaction effect shown in Table 3. A detailed description of each variable can be found in Table 1.

**Table S2 –** Calibration results table

| **Model** | ***tc*** | ***lr*** | ***bf*** | ***ss*** | **Total deviance** | **Residual deviance** | **Correlation** | **CV-Deviance** | **CV-Correlation** | **TAUC** | **cvAUC** | **ΔAUC** | ***D*^2^** |
| --- | --- | --- | --- | --- | --- | --- | --- | --- | --- | --- | --- | --- | --- |
| 1 | 1 | 0.005 | 0.5 | 25 | 0.62 | 0.58 | 0.26 | 0.58 | 0.23 | 0.69 | 0.68 | 0.01 | 7.63 |
| 2 | 1 | 0.005 | 0.5 | 50 | 0.62 | 0.57 | 0.27 | 0.58 | 0.24 | 0.70 | 0.68 | 0.02 | 8.50 |
| 3 | 1 | 0.005 | 0.7 | 25 | 0.62 | 0.58 | 0.26 | 0.58 | 0.23 | 0.70 | 0.68 | 0.01 | 7.66 |
| 4 | 1 | 0.005 | 0.7 | 50 | 0.62 | 0.57 | 0.27 | 0.58 | 0.24 | 0.70 | 0.68 | 0.02 | 8.40 |
| 5 | 1 | 0.005 | 0.9 | 25 | 0.62 | 0.58 | 0.25 | 0.59 | 0.23 | 0.69 | 0.68 | 0.01 | 7.41 |
| 6 | 1 | 0.005 | 0.9 | 50 | 0.62 | 0.57 | 0.27 | 0.58 | 0.24 | 0.70 | 0.68 | 0.02 | 8.32 |
| 7 | 1 | 0.001 | 0.5 | 25 | 0.62 | 0.58 | 0.24 | 0.59 | 0.22 | 0.69 | 0.68 | 0.01 | 6.91 |
| 8 | 1 | 0.001 | 0.5 | 50 | 0.62 | 0.58 | 0.26 | 0.58 | 0.23 | 0.70 | 0.68 | 0.01 | 7.73 |
| 9 | 1 | 0.001 | 0.7 | 25 | 0.62 | 0.58 | 0.24 | 0.59 | 0.22 | 0.69 | 0.68 | 0.01 | 6.83 |
| 10 | 1 | 0.001 | 0.7 | 50 | 0.62 | 0.58 | 0.26 | 0.58 | 0.23 | 0.70 | 0.68 | 0.01 | 7.62 |
| 11 | 1 | 0.001 | 0.9 | 25 | 0.62 | 0.58 | 0.24 | 0.59 | 0.22 | 0.69 | 0.68 | 0.01 | 6.65 |
| 12 | 1 | 0.001 | 0.9 | 50 | 0.62 | 0.58 | 0.25 | 0.58 | 0.23 | 0.70 | 0.68 | 0.01 | 7.44 |
| 13 | 1 | 0.0005 | 0.5 | 25 | 0.62 | 0.60 | 0.19 | 0.60 | 0.18 | 0.67 | 0.66 | 0.01 | 3.97 |
| 14 | 1 | 0.0005 | 0.5 | 50 | 0.62 | 0.59 | 0.22 | 0.59 | 0.20 | 0.68 | 0.67 | 0.01 | 5.38 |
| 15 | 1 | 0.0005 | 0.7 | 25 | 0.62 | 0.60 | 0.19 | 0.60 | 0.18 | 0.67 | 0.66 | 0.01 | 3.73 |
| 16 | 1 | 0.0005 | 0.7 | 50 | 0.62 | 0.59 | 0.22 | 0.60 | 0.20 | 0.68 | 0.67 | 0.01 | 5.15 |
| 17 | 1 | 0.0005 | 0.9 | 25 | 0.62 | 0.60 | 0.19 | 0.60 | 0.17 | 0.66 | 0.66 | 0.01 | 3.56 |
| 18 | 1 | 0.0005 | 0.9 | 50 | 0.62 | 0.59 | 0.21 | 0.60 | 0.19 | 0.68 | 0.67 | 0.01 | 4.96 |
| 19 | 1 | 0.0001 | 0.5 | 25 | 0.62 | 0.62 | 0.14 | 0.62 | 0.12 | 0.63 | 0.62 | 0.00 | 0.12 |
| 20 | 1 | 0.0001 | 0.5 | 50 | 0.62 | 0.62 | 0.14 | 0.62 | 0.13 | 0.63 | 0.63 | 0.00 | 0.24 |
| 21 | 1 | 0.0001 | 0.7 | 25 | 0.62 | 0.62 | 0.14 | 0.62 | 0.12 | 0.60 | 0.61 | 0.00 | 0.12 |
| 22 | 1 | 0.0001 | 0.7 | 50 | 0.62 | 0.62 | 0.14 | 0.62 | 0.13 | 0.60 | 0.62 | -0.02 | 0.22 |
| 23 | 1 | 0.0001 | 0.9 | 25 | 0.62 | 0.62 | 0.13 | 0.62 | 0.12 | 0.60 | 0.60 | 0.00 | 0.12 |
| 24 | 1 | 0.0001 | 0.9 | 50 | 0.62 | 0.62 | 0.13 | 0.62 | 0.11 | 0.60 | 0.60 | 0.00 | 0.22 |
| 25 | 2 | 0.005 | 0.5 | 25 | 0.62 | 0.49 | 0.46 | 0.53 | 0.39 | 0.81 | 0.76 | 0.05 | 20.83 |
| 26 | 2 | 0.005 | 0.5 | 50 | 0.62 | 0.46 | 0.53 | 0.51 | 0.41 | 0.85 | 0.77 | 0.08 | 26.94 |
| 27 | 2 | 0.005 | 0.7 | 25 | 0.62 | 0.49 | 0.47 | 0.53 | 0.39 | 0.81 | 0.76 | 0.05 | 21.37 |
| 28 | 2 | 0.005 | 0.7 | 50 | 0.62 | 0.44 | 0.54 | 0.51 | 0.42 | 0.86 | 0.78 | 0.08 | 28.83 |
| 29 | 2 | 0.005 | 0.9 | 25 | 0.62 | 0.49 | 0.46 | 0.53 | 0.39 | 0.81 | 0.76 | 0.05 | 21.09 |
| 30 | 2 | 0.005 | 0.9 | 50 | 0.62 | 0.44 | 0.55 | 0.51 | 0.42 | 0.87 | 0.79 | 0.08 | 29.08 |
| 31 | 2 | 0.001 | 0.5 | 25 | 0.62 | 0.52 | 0.41 | 0.54 | 0.36 | 0.77 | 0.74 | 0.03 | 16.41 |
| 32 | 2 | 0.001 | 0.5 | 50 | 0.62 | 0.49 | 0.46 | 0.53 | 0.39 | 0.81 | 0.75 | 0.05 | 21.01 |
| 33 | 2 | 0.001 | 0.7 | 25 | 0.62 | 0.52 | 0.41 | 0.54 | 0.36 | 0.77 | 0.74 | 0.03 | 16.44 |
| 34 | 2 | 0.001 | 0.7 | 50 | 0.62 | 0.49 | 0.47 | 0.53 | 0.39 | 0.81 | 0.76 | 0.05 | 21.24 |
| 35 | 2 | 0.001 | 0.9 | 25 | 0.62 | 0.52 | 0.41 | 0.54 | 0.36 | 0.77 | 0.73 | 0.03 | 16.10 |
| 36 | 2 | 0.001 | 0.9 | 50 | 0.62 | 0.50 | 0.46 | 0.53 | 0.38 | 0.80 | 0.75 | 0.05 | 20.48 |
| 37 | 2 | 0.0005 | 0.5 | 25 | 0.62 | 0.56 | 0.33 | 0.57 | 0.30 | 0.72 | 0.71 | 0.01 | 9.91 |
| 38 | 2 | 0.0005 | 0.5 | 50 | 0.62 | 0.55 | 0.36 | 0.56 | 0.32 | 0.73 | 0.72 | 0.02 | 12.23 |
| 39 | 2 | 0.0005 | 0.7 | 25 | 0.62 | 0.56 | 0.32 | 0.57 | 0.30 | 0.72 | 0.71 | 0.01 | 9.70 |
| 40 | 2 | 0.0005 | 0.7 | 50 | 0.62 | 0.55 | 0.36 | 0.56 | 0.32 | 0.73 | 0.72 | 0.02 | 12.21 |
| 41 | 2 | 0.0005 | 0.9 | 25 | 0.62 | 0.57 | 0.32 | 0.57 | 0.29 | 0.72 | 0.70 | 0.01 | 9.52 |
| 42 | 2 | 0.0005 | 0.9 | 50 | 0.62 | 0.55 | 0.35 | 0.56 | 0.32 | 0.73 | 0.71 | 0.02 | 11.59 |
| 43 | 2 | 0.0001 | 0.5 | 25 | 0.62 | 0.62 | 0.21 | 0.62 | 0.19 | 0.66 | 0.66 | 0.01 | 0.63 |
| 44 | 2 | 0.0001 | 0.5 | 50 | 0.62 | 0.60 | 0.25 | 0.60 | 0.24 | 0.69 | 0.68 | 0.01 | 3.98 |
| 45 | 2 | 0.0001 | 0.7 | 25 | 0.62 | 0.62 | 0.20 | 0.62 | 0.18 | 0.66 | 0.65 | 0.01 | 0.51 |
| 46 | 2 | 0.0001 | 0.7 | 50 | 0.62 | 0.60 | 0.24 | 0.60 | 0.23 | 0.69 | 0.68 | 0.01 | 3.92 |
| 47 | 2 | 0.0001 | 0.9 | 25 | 0.62 | 0.62 | 0.19 | 0.62 | 0.18 | 0.64 | 0.63 | 0.01 | 0.63 |
| 48 | 2 | 0.0001 | 0.9 | 50 | 0.62 | 0.60 | 0.24 | 0.60 | 0.23 | 0.68 | 0.67 | 0.01 | 3.90 |
| 49 | 3 | 0.005 | 0.5 | 25 | 0.62 | 0.41 | 0.61 | 0.49 | 0.46 | 0.90 | 0.81 | 0.09 | 34.92 |
| 50 | 3 | 0.005 | 0.5 | 50 | 0.62 | 0.34 | 0.71 | 0.46 | 0.49 | 0.95 | 0.84 | 0.11 | 46.07 |
| 51 | 3 | 0.005 | 0.7 | 25 | 0.62 | 0.40 | 0.63 | 0.48 | 0.47 | 0.91 | 0.82 | 0.10 | 36.52 |
| 52 | 3 | 0.005 | 0.7 | 50 | 0.62 | 0.32 | 0.73 | 0.46 | 0.51 | 0.96 | 0.84 | 0.12 | 49.16 |
| 53 | 3 | 0.005 | 0.9 | 25 | 0.62 | 0.40 | 0.61 | 0.48 | 0.47 | 0.90 | 0.81 | 0.09 | 35.18 |
| 54 | 3 | 0.005 | 0.9 | 50 | 0.62 | 0.33 | 0.72 | 0.46 | 0.50 | 0.95 | 0.84 | 0.11 | 47.13 |
| 55 | 3 | 0.001 | 0.5 | 25 | 0.62 | 0.46 | 0.52 | 0.51 | 0.43 | 0.85 | 0.78 | 0.06 | 25.60 |
| 56 | 3 | 0.001 | 0.5 | 50 | 0.62 | 0.40 | 0.62 | 0.49 | 0.46 | 0.91 | 0.81 | 0.09 | 35.68 |
| 57 | 3 | 0.001 | 0.7 | 25 | 0.62 | 0.46 | 0.52 | 0.51 | 0.43 | 0.85 | 0.78 | 0.06 | 25.78 |
| 58 | 3 | 0.001 | 0.7 | 50 | 0.62 | 0.39 | 0.63 | 0.48 | 0.47 | 0.92 | 0.82 | 0.10 | 37.28 |
| 59 | 3 | 0.001 | 0.9 | 25 | 0.62 | 0.46 | 0.52 | 0.51 | 0.43 | 0.85 | 0.78 | 0.06 | 25.91 |
| 60 | 3 | 0.001 | 0.9 | 50 | 0.62 | 0.40 | 0.62 | 0.48 | 0.47 | 0.91 | 0.82 | 0.09 | 36.11 |
| 61 | 3 | 0.0005 | 0.5 | 25 | 0.62 | 0.54 | 0.38 | 0.55 | 0.35 | 0.74 | 0.72 | 0.02 | 13.03 |
| 62 | 3 | 0.0005 | 0.5 | 50 | 0.62 | 0.52 | 0.42 | 0.54 | 0.38 | 0.77 | 0.74 | 0.03 | 16.97 |
| 63 | 3 | 0.0005 | 0.7 | 25 | 0.62 | 0.54 | 0.38 | 0.55 | 0.34 | 0.74 | 0.72 | 0.02 | 12.80 |
| 64 | 3 | 0.0005 | 0.7 | 50 | 0.62 | 0.52 | 0.43 | 0.54 | 0.38 | 0.77 | 0.74 | 0.03 | 16.98 |
| 65 | 3 | 0.0005 | 0.9 | 25 | 0.62 | 0.55 | 0.37 | 0.56 | 0.34 | 0.74 | 0.72 | 0.02 | 12.60 |
| 66 | 3 | 0.0005 | 0.9 | 50 | 0.62 | 0.52 | 0.42 | 0.54 | 0.38 | 0.77 | 0.74 | 0.03 | 16.88 |
| 67 | 3 | 0.0001 | 0.5 | 25 | 0.62 | 0.61 | 0.23 | 0.61 | 0.22 | 0.67 | 0.66 | 0.01 | 2.30 |
| 68 | 3 | 0.0001 | 0.5 | 50 | 0.62 | 0.58 | 0.30 | 0.59 | 0.28 | 0.70 | 0.69 | 0.01 | 6.57 |
| 69 | 3 | 0.0001 | 0.7 | 25 | 0.62 | 0.61 | 0.22 | 0.61 | 0.21 | 0.67 | 0.66 | 0.00 | 1.83 |
| 70 | 3 | 0.0001 | 0.7 | 50 | 0.62 | 0.58 | 0.30 | 0.59 | 0.28 | 0.70 | 0.69 | 0.01 | 6.45 |
| 71 | 3 | 0.0001 | 0.9 | 25 | 0.62 | 0.61 | 0.21 | 0.61 | 0.20 | 0.66 | 0.66 | 0.00 | 1.70 |
| 72 | 3 | 0.0001 | 0.9 | 50 | 0.62 | 0.59 | 0.30 | 0.59 | 0.28 | 0.70 | 0.69 | 0.01 | 6.36 |
| 73 | 4 | 0.005 | 0.5 | 25 | 0.62 | 0.33 | 0.72 | 0.46 | 0.51 | 0.95 | 0.84 | 0.11 | 46.47 |
| 74 | 4 | 0.005 | 0.5 | 50 | 0.62 | 0.26 | 0.81 | 0.43 | 0.54 | 0.98 | 0.86 | 0.12 | 58.71 |
| 75 | 4 | 0.005 | 0.7 | 25 | 0.62 | 0.34 | 0.71 | 0.46 | 0.51 | 0.95 | 0.84 | 0.11 | 46.00 |
| 76 | 4 | 0.005 | 0.7 | 50 | 0.62 | 0.26 | 0.81 | 0.44 | 0.54 | 0.98 | 0.86 | 0.12 | 58.35 |
| 77 | 4 | 0.005 | 0.9 | 25 | 0.62 | 0.33 | 0.72 | 0.45 | 0.51 | 0.95 | 0.84 | 0.11 | 46.58 |
| 78 | 4 | 0.005 | 0.9 | 50 | 0.62 | 0.27 | 0.79 | 0.44 | 0.53 | 0.98 | 0.86 | 0.12 | 56.24 |
| 79 | 4 | 0.001 | 0.5 | 25 | 0.62 | 0.40 | 0.62 | 0.48 | 0.48 | 0.91 | 0.82 | 0.09 | 35.28 |
| 80 | 4 | 0.001 | 0.5 | 50 | 0.62 | 0.33 | 0.72 | 0.46 | 0.51 | 0.95 | 0.84 | 0.11 | 47.03 |
| 81 | 4 | 0.001 | 0.7 | 25 | 0.62 | 0.40 | 0.63 | 0.48 | 0.48 | 0.91 | 0.82 | 0.09 | 35.97 |
| 82 | 4 | 0.001 | 0.7 | 50 | 0.62 | 0.32 | 0.73 | 0.45 | 0.52 | 0.96 | 0.85 | 0.11 | 48.23 |
| 83 | 4 | 0.001 | 0.9 | 25 | 0.62 | 0.41 | 0.61 | 0.48 | 0.47 | 0.90 | 0.82 | 0.08 | 34.73 |
| 84 | 4 | 0.001 | 0.9 | 50 | 0.62 | 0.33 | 0.72 | 0.46 | 0.51 | 0.95 | 0.84 | 0.11 | 46.42 |
| 85 | 4 | 0.0005 | 0.5 | 25 | 0.62 | 0.53 | 0.41 | 0.54 | 0.37 | 0.76 | 0.74 | 0.03 | 15.55 |
| 86 | 4 | 0.0005 | 0.5 | 50 | 0.62 | 0.49 | 0.48 | 0.52 | 0.41 | 0.82 | 0.77 | 0.05 | 21.76 |
| 87 | 4 | 0.0005 | 0.7 | 25 | 0.62 | 0.53 | 0.42 | 0.54 | 0.37 | 0.76 | 0.74 | 0.03 | 15.63 |
| 88 | 4 | 0.0005 | 0.7 | 50 | 0.62 | 0.49 | 0.49 | 0.52 | 0.42 | 0.82 | 0.77 | 0.05 | 21.96 |
| 89 | 4 | 0.0005 | 0.9 | 25 | 0.62 | 0.53 | 0.41 | 0.54 | 0.37 | 0.76 | 0.73 | 0.02 | 15.04 |
| 90 | 4 | 0.0005 | 0.9 | 50 | 0.62 | 0.49 | 0.48 | 0.52 | 0.41 | 0.81 | 0.76 | 0.05 | 21.17 |
| 91 | 4 | 0.0001 | 0.5 | 25 | 0.62 | 0.60 | 0.28 | 0.60 | 0.26 | 0.69 | 0.68 | 0.01 | 4.64 |
| 92 | 4 | 0.0001 | 0.5 | 50 | 0.62 | 0.57 | 0.33 | 0.58 | 0.31 | 0.71 | 0.70 | 0.01 | 8.14 |
| 93 | 4 | 0.0001 | 0.7 | 25 | 0.62 | 0.59 | 0.28 | 0.60 | 0.26 | 0.69 | 0.68 | 0.01 | 4.78 |
| 94 | 4 | 0.0001 | 0.7 | 50 | 0.62 | 0.58 | 0.33 | 0.58 | 0.30 | 0.71 | 0.70 | 0.01 | 7.94 |
| 95 | 4 | 0.0001 | 0.9 | 25 | 0.62 | 0.59 | 0.27 | 0.60 | 0.26 | 0.69 | 0.68 | 0.01 | 4.89 |
| 96 | 4 | 0.0001 | 0.9 | 50 | 0.62 | 0.58 | 0.33 | 0.58 | 0.30 | 0.71 | 0.70 | 0.01 | 7.85 |
| 97 | 5 | 0.005 | 0.5 | 25 | 0.62 | 0.28 | 0.78 | 0.44 | 0.54 | 0.98 | 0.86 | 0.11 | 54.68 |
| 98 | 5 | 0.005 | 0.5 | 50 | 0.62 | 0.22 | 0.86 | 0.42 | 0.56 | 0.99 | 0.88 | 0.12 | 64.72 |
| 99 | 5 | 0.005 | 0.7 | 25 | 0.62 | 0.28 | 0.79 | 0.43 | 0.54 | 0.98 | 0.86 | 0.11 | 55.67 |
| 100 | 5 | 0.005 | 0.7 | 50 | 0.62 | 0.22 | 0.86 | 0.42 | 0.56 | 0.99 | 0.88 | 0.12 | 65.19 |
| 101 | 5 | 0.005 | 0.9 | 25 | 0.62 | 0.28 | 0.79 | 0.44 | 0.54 | 0.97 | 0.86 | 0.11 | 55.15 |
| 102 | 5 | 0.005 | 0.9 | 50 | 0.62 | 0.22 | 0.86 | 0.42 | 0.56 | 0.99 | 0.87 | 0.12 | 64.87 |
| 103 | 5 | 0.001 | 0.5 | 25 | 0.62 | 0.36 | 0.68 | 0.46 | 0.51 | 0.94 | 0.84 | 0.10 | 42.25 |
| 104 | 5 | 0.001 | 0.5 | 50 | 0.62 | 0.28 | 0.79 | 0.43 | 0.54 | 0.98 | 0.87 | 0.11 | 55.87 |
| 105 | 5 | 0.001 | 0.7 | 25 | 0.62 | 0.35 | 0.69 | 0.46 | 0.51 | 0.94 | 0.84 | 0.10 | 43.46 |
| 106 | 5 | 0.001 | 0.7 | 50 | 0.62 | 0.27 | 0.80 | 0.43 | 0.55 | 0.98 | 0.86 | 0.11 | 56.75 |
| 107 | 5 | 0.001 | 0.9 | 25 | 0.62 | 0.37 | 0.68 | 0.47 | 0.50 | 0.93 | 0.83 | 0.10 | 41.15 |
| 108 | 5 | 0.001 | 0.9 | 50 | 0.62 | 0.28 | 0.78 | 0.44 | 0.54 | 0.97 | 0.86 | 0.12 | 54.60 |
| 109 | 5 | 0.0005 | 0.5 | 25 | 0.62 | 0.51 | 0.44 | 0.53 | 0.39 | 0.78 | 0.75 | 0.03 | 17.85 |
| 110 | 5 | 0.0005 | 0.5 | 50 | 0.62 | 0.45 | 0.54 | 0.50 | 0.45 | 0.86 | 0.79 | 0.06 | 27.21 |
| 111 | 5 | 0.0005 | 0.7 | 25 | 0.62 | 0.51 | 0.44 | 0.53 | 0.39 | 0.78 | 0.75 | 0.03 | 17.65 |
| 112 | 5 | 0.0005 | 0.7 | 50 | 0.62 | 0.46 | 0.54 | 0.50 | 0.45 | 0.86 | 0.79 | 0.06 | 26.93 |
| 113 | 5 | 0.0005 | 0.9 | 25 | 0.62 | 0.51 | 0.44 | 0.53 | 0.39 | 0.78 | 0.75 | 0.03 | 17.78 |
| 114 | 5 | 0.0005 | 0.9 | 50 | 0.62 | 0.46 | 0.53 | 0.51 | 0.44 | 0.85 | 0.79 | 0.06 | 26.05 |
| 115 | 5 | 0.0001 | 0.5 | 25 | 0.62 | 0.59 | 0.31 | 0.59 | 0.29 | 0.70 | 0.69 | 0.01 | 6.04 |
| 116 | 5 | 0.0001 | 0.5 | 50 | 0.62 | 0.57 | 0.35 | 0.57 | 0.33 | 0.72 | 0.71 | 0.01 | 9.37 |
| 117 | 5 | 0.0001 | 0.7 | 25 | 0.62 | 0.59 | 0.30 | 0.59 | 0.28 | 0.70 | 0.69 | 0.01 | 6.10 |
| 118 | 5 | 0.0001 | 0.7 | 50 | 0.62 | 0.57 | 0.35 | 0.57 | 0.32 | 0.72 | 0.71 | 0.02 | 9.16 |
| 119 | 5 | 0.0001 | 0.9 | 25 | 0.62 | 0.59 | 0.30 | 0.59 | 0.29 | 0.70 | 0.69 | 0.01 | 6.18 |
| 120 | 5 | 0.0001 | 0.9 | 50 | 0.62 | 0.57 | 0.35 | 0.57 | 0.32 | 0.72 | 0.71 | 0.02 | 9.10 |
| 121 | 6 | 0.005 | 0.5 | 25 | 0.62 | 0.24 | 0.83 | 0.42 | 0.56 | 0.99 | 0.87 | 0.11 | 61.32 |
| 122 | 6 | 0.005 | 0.5 | 50 | 0.62 | 0.19 | 0.89 | 0.41 | 0.58 | 1.00 | 0.89 | 0.11 | 69.83 |
| 123 | 6 | 0.005 | 0.7 | 25 | 0.62 | 0.24 | 0.84 | 0.42 | 0.56 | 0.99 | 0.87 | 0.11 | 61.62 |
| **124** | **6** | **0.005** | **0.7** | **50** | **0.62** | **0.18** | **0.90** | **0.41** | **0.58** | **1.00** | **0.89** | **0.11** | **71.97** |
| 125 | 6 | 0.005 | 0.9 | 25 | 0.62 | 0.25 | 0.82 | 0.42 | 0.56 | 0.98 | 0.87 | 0.11 | 59.91 |
| 126 | 6 | 0.005 | 0.9 | 50 | 0.62 | 0.19 | 0.89 | 0.41 | 0.58 | 1.00 | 0.88 | 0.11 | 69.72 |
| 127 | 6 | 0.001 | 0.5 | 25 | 0.62 | 0.32 | 0.74 | 0.44 | 0.53 | 0.96 | 0.86 | 0.11 | 48.44 |
| 128 | 6 | 0.001 | 0.5 | 50 | 0.62 | 0.24 | 0.84 | 0.42 | 0.56 | 0.99 | 0.88 | 0.11 | 61.39 |
| 129 | 6 | 0.001 | 0.7 | 25 | 0.62 | 0.32 | 0.74 | 0.45 | 0.53 | 0.96 | 0.85 | 0.11 | 48.29 |
| 130 | 6 | 0.001 | 0.7 | 50 | 0.62 | 0.24 | 0.84 | 0.42 | 0.56 | 0.99 | 0.88 | 0.11 | 61.59 |
| 131 | 6 | 0.001 | 0.9 | 25 | 0.62 | 0.33 | 0.72 | 0.45 | 0.52 | 0.95 | 0.85 | 0.10 | 46.64 |
| 132 | 6 | 0.001 | 0.9 | 50 | 0.62 | 0.24 | 0.83 | 0.42 | 0.56 | 0.99 | 0.87 | 0.11 | 60.91 |
| 133 | 6 | 0.0005 | 0.5 | 25 | 0.62 | 0.49 | 0.48 | 0.52 | 0.42 | 0.81 | 0.77 | 0.05 | 20.96 |
| 134 | 6 | 0.0005 | 0.5 | 50 | 0.62 | 0.43 | 0.59 | 0.49 | 0.47 | 0.89 | 0.81 | 0.08 | 31.16 |
| 135 | 6 | 0.0005 | 0.7 | 25 | 0.62 | 0.50 | 0.48 | 0.52 | 0.41 | 0.80 | 0.76 | 0.04 | 20.23 |
| 136 | 6 | 0.0005 | 0.7 | 50 | 0.62 | 0.43 | 0.59 | 0.49 | 0.47 | 0.88 | 0.81 | 0.08 | 30.90 |
| 137 | 6 | 0.0005 | 0.9 | 25 | 0.62 | 0.50 | 0.47 | 0.52 | 0.41 | 0.80 | 0.76 | 0.04 | 19.98 |
| 138 | 6 | 0.0005 | 0.9 | 50 | 0.62 | 0.43 | 0.59 | 0.49 | 0.47 | 0.88 | 0.81 | 0.07 | 31.48 |
| 139 | 6 | 0.0001 | 0.5 | 25 | 0.62 | 0.58 | 0.33 | 0.58 | 0.31 | 0.71 | 0.70 | 0.01 | 6.97 |
| 140 | 6 | 0.0001 | 0.5 | 50 | 0.62 | 0.56 | 0.37 | 0.57 | 0.34 | 0.73 | 0.72 | 0.02 | 10.48 |
| 141 | 6 | 0.0001 | 0.7 | 25 | 0.62 | 0.58 | 0.32 | 0.59 | 0.30 | 0.70 | 0.69 | 0.01 | 6.86 |
| 142 | 6 | 0.0001 | 0.7 | 50 | 0.62 | 0.56 | 0.37 | 0.57 | 0.34 | 0.73 | 0.71 | 0.02 | 10.28 |
| 143 | 6 | 0.0001 | 0.9 | 25 | 0.62 | 0.58 | 0.32 | 0.59 | 0.30 | 0.70 | 0.69 | 0.01 | 6.86 |
| 144 | 6 | 0.0001 | 0.9 | 50 | 0.62 | 0.56 | 0.36 | 0.57 | 0.34 | 0.73 | 0.71 | 0.02 | 10.13 |
